# Host-specific ubiquitination of prM orchestrates ESCRT recruitment to mediate efficient Japanese Encephalitis Virus assembly in vertebrates

**DOI:** 10.1371/journal.ppat.1014426

**Published:** 2026-07-08

**Authors:** Chenxi Li, Wenzhuang Guo, Wen Zhao, Linjie Zhang, Chenyang Tang, Jingjing Li, Jing Shi, Mingan Sun, Yanhua Li

**Affiliations:** 1 College of Veterinary Medicine, Yangzhou University, Yangzhou, Jiangsu, China; 2 Comparative Medicine Research Institute, Yangzhou University, Yangzhou, Jiangsu, China; 3 Jiangsu Co-innovation Center for Prevention and Control of Important Animal Infectious Diseases and Zoonoses, Yangzhou, Jiangsu, China; 4 Department of Agricultural and Animal Husbandry Engineering, Cangzhou Technical College, Cangzhou, Hebei, China; 5 The Interdisciplinary Center for Zoonotic Diseases and Biosafety, Yangzhou University, Yangzhou, Jiangsu, China; The University of Texas Medical Branch at Galveston, UNITED STATES OF AMERICA

## Abstract

Mosquito-borne orthoflavivirus, such as Japanese encephalitis virus (JEV), Dengue virus (DENV), and Zika virus (ZIKV), pose a serious global health threat. As obligate intracellular parasites, they often hijack the host ubiquitin system to modify their own proteins, thereby regulating the viral life cycle, host adaptation, transmission, and pathogenesis. Despite its critical roles, the precise molecular mechanisms and functional significance of viral protein ubiquitination in orthoflavivirus infection remain incompletely understood. Here, we identify JEV prM as a novel target for host-specific ubiquitination, which occurs exclusively in vertebrate hosts but not in mosquitoes. Ubiquitin conjugation at the evolutionarily conserved lysine residues (K107/108/116) in multiple mosquito-borne orthoflaviviruses (USUV, MVEV, and WNV) confers differential adaptation between vertebrate hosts and mosquito vectors. Mechanistically, prM ubiquitination serves as a recruitment signal for the ESCRT-I subunit TSG101, an early-acting component of the ESCRT machinery, which in turn serves as an adaptor to recruit downstream ESCRT components (VPS28, CHMP2A, and CHMP4B), thereby driving viral particle budding. These findings elucidate a novel mechanism by which viral protein ubiquitination regulates JEV infection and host adaptation, and provide important insights into the adaptive evolution of orthoflaviviruses across different hosts and vectors.

## Author summary

As a mosquito-borne zoonotic orthoflavivirus, JEV is the leading cause of viral encephalitis in many countries of Asia with an estimated 100,000 clinical human cases. In orthoflaviviruses, the ubiquitination of viral proteins has diverse functions in the viral life cycle, pathogenesis, and transmission. Here, we identify JEV prM as a novel target for host-specific ubiquitination, which occurs exclusively in vertebrate hosts and confers differential adaptation between vertebrate hosts and the mosquito vector. Moreover, we revealed the mechanism, by which prM ubiquitination recruits the ESCRT-I subunit TSG101, which bridges the recruitment of downstream ESCRT components to facilitate viral budding. This work disclosed a novel role of viral protein ubiquitination in regulating JEV replication and host adaptation, providing a potential therapeutic target to interrupt orthoflaviviruses infection and transmission.

## Introduction

The genus *Orthoflavivirus* within the family *Flaviviridae* encompasses a variety of highly pathogenic viruses, including Zika virus (ZIKV), West Nile virus (WNV), Tick-borne encephalitis virus (TBEV), Dengue virus (DENV), and Japanese encephalitis virus (JEV) [1,2]. These viruses are transmitted by arthropod vectors (ticks and mosquitoes), and pose a serious threat to human health [3,4]. JEV is an important mosquito-borne zoonotic pathogen that cause severe viral encephalitis in humans, horses, and piglets and reproductive disorders in breeding pigs [5–7]. According to the World Health Organization (WHO), the estimated annual incidence of human JEV infection cases is in the range of 30,000–50,000, with approximately 10,000–15,000 fatalities and up to 50% of survivors suffering from permanent neurological or psychiatric sequelae [8,9]. Despite being vaccine-preventable, it remains endemic in 24 countries and territories throughout Asia and Oceania [10]. In nature, JEV is maintained in a transmission cycle between mosquito vectors and vertebrate-amplifying hosts (birds and pigs) [11,12]. As the amplifying hosts, infected birds and pigs could develop a viremia that is high enough to infect mosquitoes, thereby playing a critical role in the maintenance and transmission of the JEV [10,12–14]. Humans and horses are considered the dead-end hosts of JEV.

Like other orthoflaviviruses, JEV is an enveloped virus containing a positive-sense, single-stranded RNA genome of approximately 11 kb in length [15]. The genomic RNA contains a single open-reading frame (ORF) flanked by a 5’ untranslated region (UTR) and a 3’ UTR. The ORF encodes a single polyprotein which is post-translationally processed by viral and host proteases to generate three structural proteins [capsid (C), pre-membrane/membrane (prM/M), and envelope (E)] and seven nonstructural proteins (NS1, NS2A, NS2B, NS3, NS4A, NS4B, and NS5) [15,16]. The structural proteins, together with genomic RNA, constitute the components of virions, while non-structural proteins are involved in viral RNA replication, virion assembly, and host immune response evasion. The replication and assembly of orthoflavivirus occur at the endoplasmic reticulum (ER), facilitated by a dramatic rearrangement of the membrane structure that generates a specific compartment generally referred to as the viral replication organelle [17,18]. Orthoflavivirus genome replication induces the formation of vesicle compartments within the ER lumen, with their outer membranes connected to the ER via a neck-like structure [18]. The newly synthesized viral genome is assembled on the ER membrane and buds into the ER lumen to form a viral particle [18]. This budding process is primarily driven by the viral structural proteins prM and E. Wherein, prM is a transmembrane glycoprotein residing in the ER that crucially orchestrates both virion assembly and viral particle secretion [19,20]. The orthoflavivirus prM glycoprotein interacts with E protein to form heterodimer complexes in the ER, resulting in assembly of immature virions with a rough outer surface, as prM and E form heterotrimeric spike-like structures [16]. Subsequently, immature virions translocate through the Golgi, where the low-pH environment triggers irreversible conformational changes and the cellular furin protease cleaves prM, eventually resulting in the release of the pr peptide and rendering virus particles infectious [21]. Nevertheless, the molecular mechanisms underlying the role of prM protein in the assembly of orthoflavivirus particles remain largely unknown.

Virion assembly is an important step in the life cycle of all viruses. Notably, enveloped viruses commonly acquire their envelopes at the plasma membrane by recruiting the host endosomal sorting complex required for transport (ESCRT) machinery [22,23]. In eukaryotic cells, the ESCRT pathway is a conserved membrane remodeling complex and consists of several distinct heteromeric complexes involved in membrane invagination and vesicle formation in the multivesicular bodies (MVBs) pathway [24]. During formation of the ESCRT complex, components of the early ESCRT proteins: ALG-2-interacting protein X (ALIX), ESCRT-0, ESCRT-I, or ESCRT-II proteins are recruited to ubiquitinated proteins or to proteins containing the late domain sequences, followed by the attachment of ESCRT-III proteins to elicit membrane scission [24,25]. It has been shown that many pathogenic viruses, including Ebola virus (EboV) [26], hepatitis C virus (HCV) [27], Nipah virus (NIV) [28], and human immunodeficiency virus-1 (HIV-1) [29], hijack the host ESCRT machinery to facilitate viral budding and fission from the plasma membrane. Interestingly, a distinct set of ESCRT components (TSG101, CHMP2/3, and CHMP4 proteins) also functions directly in membrane deformation during orthoflavivirus particle formation on the ER membrane [30], but the underlying mechanisms remain unclear.

As a post-translational modification that orchestrates diverse cellular functions, ubiquitination is catalyzed by a multienzyme cascade comprising E1 ubiquitin-activating enzymes, E2 ubiquitin-conjugating enzymes, and E3 ubiquitin ligases [31]. Conventionally, the canonical role of ubiquitination is to modulate protein levels by targeting substrates for degradation through the ubiquitin-proteasome system or lysosomal pathways [32]. Alternatively, the ubiquitination system could mark substrate proteins to perform diverse regulatory roles through non-degradative mechanisms, such as promoting or obstructing the interaction of proteins [33], and changing protein localization in cellular compartments [34,35]. As obligate intracellular parasites, viruses usually hijack the host ubiquitin system to modify viral proteins, thereby facilitating various stages of the viral life cycle [36,37]. In orthoflaviviruses, the ubiquitination of viral proteins has diverse functions in the viral life cycle and pathogenesis. For instance, K63- or K27- linked ubiquitination of the E protein of ZIKV and JEV facilitates viral entry by promoting the attachment to cellular receptors [6,38]. The ubiquitination of C protein is required for the efficient release of DENV genome [39], and the ubiquitination of orthoflavivirus NS2A mediated by the ER-located E3 ligase AMFR subverts ER-phagy to augment viral pathogenicity [40]. Additionally, recent researches highlighted the ubiquitination of orthoflavivirus proteins as a key determinant of viral host adaptation and transmission. The ubiquitination of NS1 has been confirmed to confer differential adaptation of ZIKV in mammalian hosts and mosquito vector [41], and the ubiquitination of NS4A driven by HRD1 determines DENV transmission from the infected vertebrates to mosquitoes [42]. Although ubiquitination of several JEV proteins, such as NS3 and NS5, has been reported [43], the functional significance of viral protein ubiquitination in JEV replication, host adaptation, and transmission remain largely unexplored. Here, we identify JEV prM as a novel target for host-specific ubiquitination, which uniquely occurs in vertebrate hosts but not in mosquitoes. This host-specific ubiquitination directly contributes to the JEV adaptation in vertebrate hosts. Mechanistically, prM ubiquitination serves as a recruitment signal for the ESCRT-I subunit TSG101, an early-acting ESCRT, which in turn acts as an adaptor to recruit downstream ESCRT components (VPS28, CHMP2A, and CHMP4B), thereby driving viral particle budding. These results elucidate a novel mechanism of ubiquitination within viral proteins in regulating JEV infection and host adaptation, and provide potential implications for the design of novel antiviral strategies against orthoflavivirus.

## Results

### Ubiquitination of the JEV prM protein occurs exclusively in vertebrate hosts, but not in mosquito vectors

**Fig 1:**
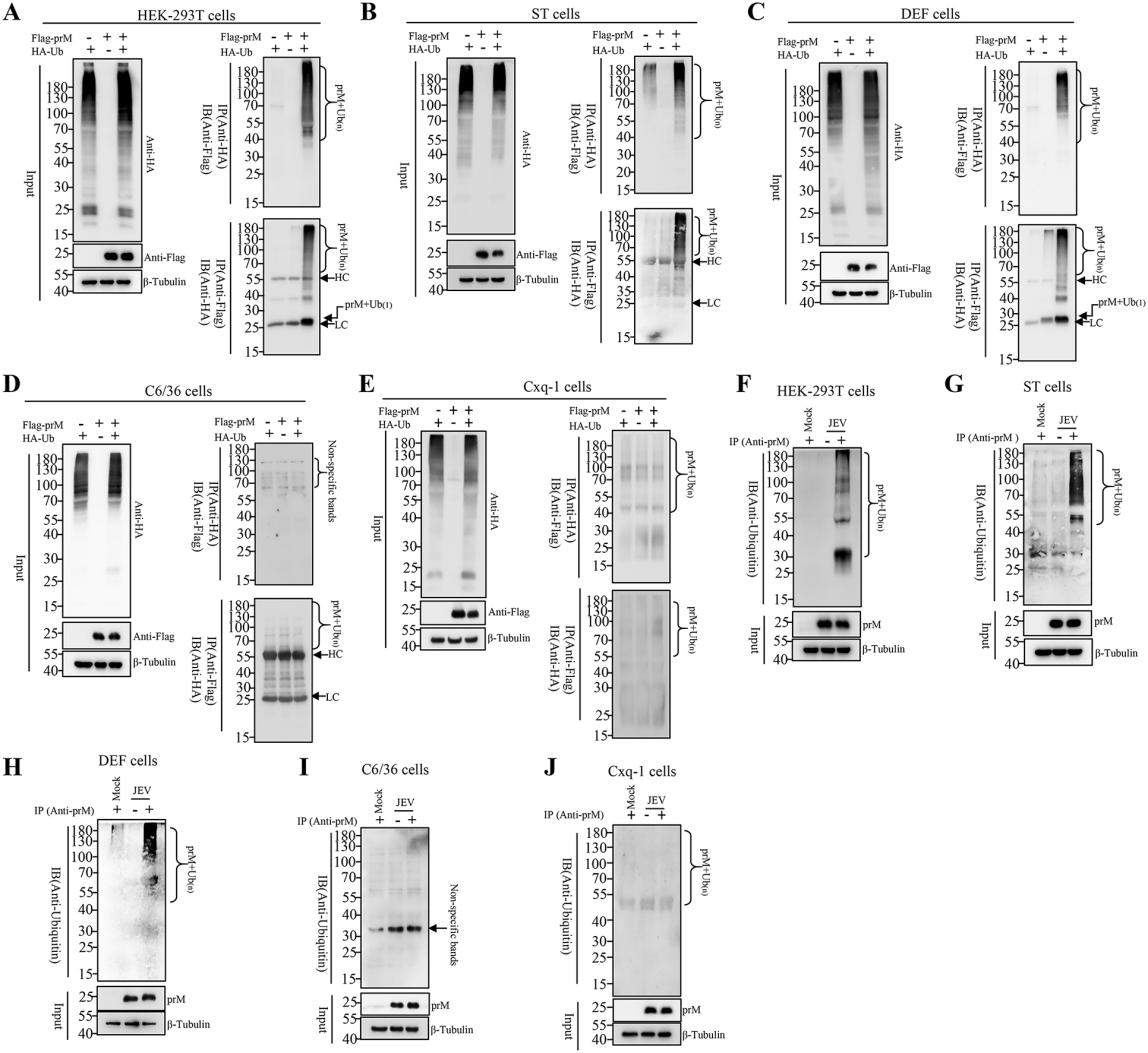
Ubiquitination of JEV prM protein occurs exclusively in vertebrate hosts, but not in mosquito vectors. **(A-E)** Ubiquitination analysis of the JEV prM protein ectopically expressed in HEK-293T **(A)**, ST **(B)**, DEF **(C)**, C6/36 **(D)**, and Cxq-1 (E) cells. Cells were respectively transfected with a plasmid expressing Flag-tagged JEV prM, along with HA-Ub plasmid or an empty vector. At 36 hpt, cell lysates were harvested for immunoprecipitation assays using anti-Flag or anti-HA magnetic beads. The ubiquitination of prM protein was analyzed by immunoblotting with an anti-HA or anti-Flag antibody. **(F-J)** Ubiquitination analysis of the JEV prM protein in JEV-infected HEK-293T **(F)**, ST **(G)**, DEF **(H)**, C6/36 **(I)**, and Cxq-1 (J) cells. Cells were either mock-infected or infected with the JEV virulent strain Beijing/2020-1 at an MOI of 1. At 24 hpi (HEK-293T, ST, and DEF) or 60 hpi (C6/36 and Cxq-1), cell lysates were harvested for immunoprecipitation assays using a prM antibody and analyzed by Western blotting using an anti-ubiquitin antibody.

Currently, multiple orthoflavivirus proteins, including C [39], E [38], NS1 [41], NS2A [40], NS4A [42], and NS5 [44], have been confirmed to undergo ubiquitination and contribute significantly to viral replication, host adaptation, and transmission. Given the involvement of prM protein in many steps of the orthoflavivirus life cycle, we therefore investigated whether prM is also ubiquitinated and aim to define the functional consequences of this modification. The ubiquitination of JEV prM was analyzed with multiple assays in cells derived from five hosts, including human embryonic kidney 293T cells (HEK-293T), swine testicular cells (ST), duck embryonic fibroblasts (DEF), *Aedes albopictus* cells (C6/36), and *Culex pipiens quinquefasciatus* cells (Cxq-1). Immunoprecipitation and Western blot analysis revealed the obvious ubiquitin conjugations on prM, evidenced by a characteristic mass shift ranging from approximately 40–180 kDa in HEK-293T, ST and DEF cells (Fig 1A-1C). Notably, the ubiquitination of prM protein was not detected in C6/36 and Cxq-1 cells (Fig 1D and 1E). To further confirm that this ubiquitination is species-specific but not cell type-specific, a panel of cell lines including mammalian BHK-21 (Baby hamster kidney) and PIEC (Porcine iliac endothelium) cells, avian DF-1 cells (chicken embryo fibroblasts), and insect cell line S2 (*Drosophila melanogaster*), was selected for ubiquitination analysis of prM protein. In line with the results in Fig 1, robust ubiquitination of prM occured exclusively in BHK-21, PIEC and DF-1 cells (S1A-1C Fig), but not in S2 cells (S1D Fig).

Further, we assessed prM ubiquitination during JEV infection. The various cell lines were infected with the JEV virulent strain Beijing/2020–1, and the ubiquitination of immunoprecipitated prM protein was evaluated with an anti-ubiquitin antibody. Consistent ubiquitination of prM was detected in mammalian HEK-293T, BHK-21, ST, and PIEC cells (Figs 1F, 1G, S1E and S1F), and avian DEF and DF-1 cells (Fig 1H and S1G), whereas prM ubiquitination was strikingly absent in either C6/36, Cxq-1, or S2 cells upon viral infection (Figs 1I, 1J and S1H). Furthermore, we analyzed the overall ubiquitination levels of C6/36 cells following viral infection and found that the global ubiquitination levels were comparable between infected and uninfected cells (S2A Fig). Meanwhile, we demonstrated that the JEV NS1 protein could undergo significant ubiquitination in mosquito cells (S2B Fig), consistent with observations for other orthoflaviviruses in mosquito cells [41]. Therefore, the ubiquitination deficiency of prM is likely attributable to an intrinsic lack of host factors essential for this modification rather than to a kinetic artifact of viral infection in mosquito cells.

Overall, these data indicate that the ubiquitination of JEV prM protein occurs exclusively in vertebrate hosts, but not in the mosquito vector.

### Lysines 107, 108, and 116 of prM are the target sites for ubiquitination

**Fig 2:**
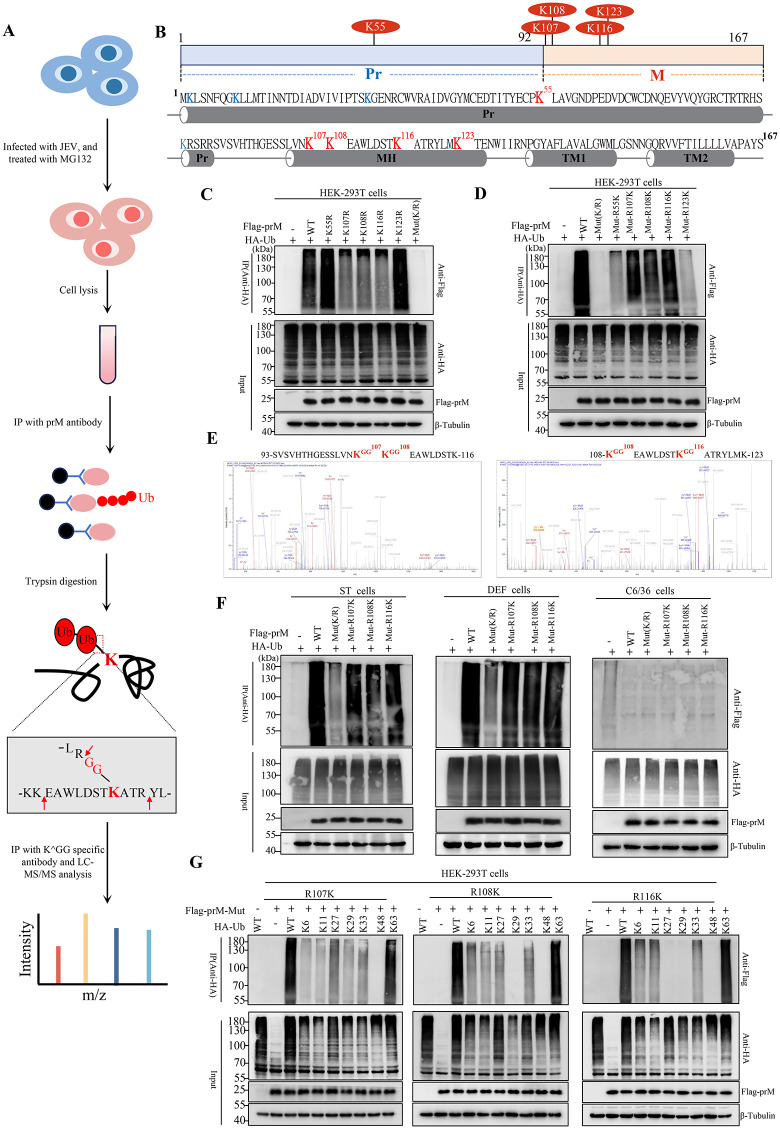
Lysines at 107, 108, and 116 of JEV prM are ubiquitination sites. **(A)** Workflow for preparing samples for K-ε-GG enrichment. BHK-21 cells were infected with the JEV virulent strain Beijing/2020-1 (MOI = 1) in the presence of 10 μM MG132. At 24 hpi, cells were harvested for immunoprecipitation using a prM antibody. Following immunoprecipitation, the immunoprecipitants were digested with LysC and trypsin. The resulting peptides were enriched using an anti-K-ε-GG antibody and analyzed by mass spectrometry. **(B)** A schematic diagram of the JEV prM protein. The lysines highlighted in red depict the location of the identified diglycylated lysines (K-ε-GG position). MH: membrane helix (MH), TM: transmembrane segment (TM). **(C, D)** Verification of ubiquitination sites of JEV prM identified by Mass spectrometry by ubiquitination analysis. HEK-293T cells were co-transfected with a plasmid expressing HA-Ub and a plasmid expressing K-to-R mutant or compensatory mutant (R-to-K) of prM. At 36 hpt, cells were lysed and analyzed by ubiquitination assay with the indicated antibodies. **(E)** Secondary mass spectrometry analysis of ubiquitination at positions K107, K108, and K116 of prM. **(F)** Ubiquitination analysis of the prM-Mut-R107K, -R108K and -R116K proteins ectopically expressed in ST, DEF, and C6/36 cells. **(G)** Identification of the ubiquitination linkage type of JEV prM protein. HEK-293T cells were transfected with a plasmid expressing a Flag-prM-Mut mutant and a plasmid expressing HA-Ub-WT or a HA-Ub mutant (Ub-K6, Ub-K11, Ub-K27, Ub-K29, Ub-K33, Ub-K48, and Ub-K63). At 24 hpi, cell lysates were harvested for immunoprecipitation assays using an anti-Flag antibody and analyzed by Western blotting using an anti-ubiquitin antibody.

Ubiquitination involves the attachment of ubiquitin to acceptor lysine residues on substrate proteins [31]. To determine the specific location of the ubiquitin-modified lysines within the JEV prM, we first explored the ubiquitination landscape of the JEV prM protein in BHK-21 cells by applying mass spectrometry analysis of immuno-purified K-ε-GG (di-glycyl)-remnant-bearing peptides (Fig 2A). JEV prM protein is composed of 167 amino acids and contains nine lysine (K) residues (Fig 2B). Mass spectrometric analysis eventually identified five lysines with di-glycyl remnants distributed across prM, of which one (K55) resided in the pr peptide, and the other four (K107, K108, K116, and K123) were situated in the membrane helix (MH) within the M protein (Fig 2B and S1 Table). To precisely define the functional sites, we created a panel of prM mutants with single lysine-to-arginine substitutions (K55R, K107R, K108R, K116R, and K123R), as well as a combined mutant [prM-Mut(K/R)] with all five substitutions. As shown in Fig 2C, the mutation of K107R, K108, and K116R markedly reduced, but could not completely abolish prM ubiquitination. In contrast, the prM-Mut(K/R) nearly eliminated the ubiquitination of prM (Fig 2C), indicating that multiple lysines on prM could be ubiquitination sites. To further clarify the contribution of individual lysine sites to prM ubiquitination, a panel of mutants carrying a single lysine and four arginines at these sites was constructed and designated as prM-Mut-R55K, -R107K, -R108K, -R116K, and -R123K. Lysines at 107, 108, and 116 robustly restored the ubiquitination of prM, whereas R55K or R123K had a minimal effect, suggesting that lysines at 107, 108, and 116 sites are the primary ubiquitination sites of prM (Fig 2D and 2E). Meanwhile, the ubiquitination status of the prM-Mut-R107K, -R108K and -R116K proteins in ST, DEF, and C6/36 cells was analyzed to investigate the functional conservation of ubiquitination sites across species. Consistently, lysines at 107, 108, and 116 sites restored the ubiquitination on prM-Mut(K/R) in ST and DEF cells, but not in C6/36 cells (Fig 2F), supporting that prM ubiquitination is a host-specific modification in vertebrates.

Ubiquitin is composed of 76 amino acid residues, including seven lysine residues. Each lysine residue could form single or mixed polyubiquitin chain linkages, thereby mediating various cellular signaling pathways [31]. The linkage type of polyubiquitin conjugation to K107, K108, and K116 residues within the prM protein was determined using wild-type HA-Ubiquitin or HA-Ubiquitin mutants. The ubiquitination of these three sites is predominantly K63-linked polyubiquitination, although other types of linkages were also detected, except K48-linked polyubiquitination (Fig 2G). Taken together, K107, K108, and K116 were identified as the critical ubiquitination sites on JEV prM and primarily targeted for K63-linked polyubiquitination.

### The ubiquitination of prM is a determinant of efficient viral replication in vertebrate cells, not in mosquito cells

**Fig 3:**
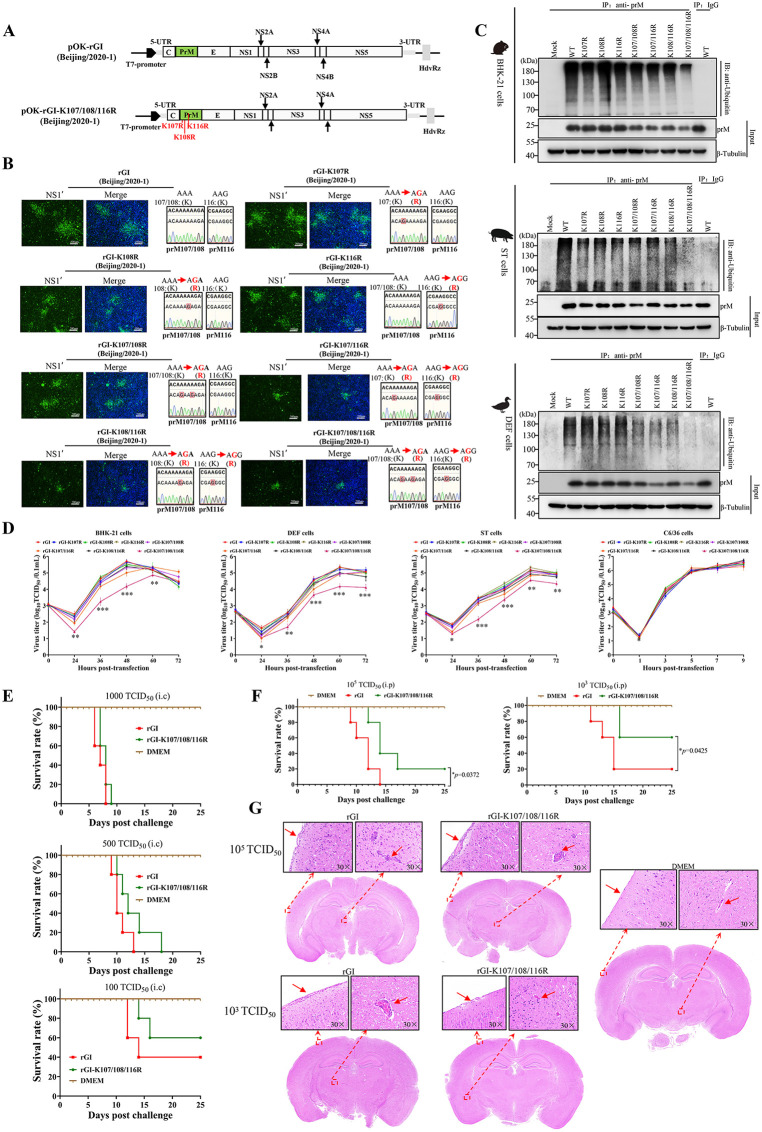
The ubiquitination of prM is important for JEV infection in vertebrate cells, but not in mosquito cells. **(A)** Schematic representation of infectious cDNA clones of the JEV Beijing/2020-1 strain and its prM mutants. **(B)** Validation of the recombinant viruses rescued by reverse genetics. BHK-21 cells transfected with the WT or mutant viral RNAs were stained with an anti-NS1’ antibody at 36 hpt. Scale bar: 200 μm. The rescued virus was serially passaged five times in BHK-21 cells and validated by Sanger sequencing. **(C)** Ubiquitination analysis of WT JEV prM protein or its mutants in JEV-infected BHK-21, ST, and DEF cells. Cells were either mock-infected or infected with WT JEV or mutants (rGI-K107R, rGI-K108R, rGI-K116R, rGI-K107/108R, rGI-K108/116R, rGI-K107/116R, rGI-K107/108/116R) at an MOI of 1. At 24 hpi, the ubiquitination of prM was assessed by immunoprecipitation with a prM antibody or control IgG, followed by immunoblotting with an anti-ubiquitin antibody. **(D)** The growth kinetics of WT JEV and its mutants in BHK-21, ST, DEF and C6/36 cells. ^[^*^]^ indicated the significant difference between rGI and rGI-K107/108/116R. **(E, F)** The survival curve of mice (n = 5) intracerebrally inoculated with the WT rGI and rGI-K107/108/116R at doses of 100, 500, and 1000 TCID_50_ **(E)**, or intraperitoneally inoculated with these two viruses at doses of 10^5^ and 10^3^ TCID_50_ **(F)**. **(G)** The pathological changes in the brain tissues were examined by H&E staining, represented by red arrows. Error bars present as the mean ± SD (n = 3 independent experiments) **(D)**; Statistical analysis was performed using one-way ANOVA with Tukey’s multiple comparisons test (D) or Kaplan–Meier analysis **(F)**, *p < 0.05, **p < 0.01, ***p < 0.001.

The prM protein is involved in viral assembly, virion maturation and viral virulence of JEV [19,21,45]. To investigate the function of the lysine residues at 107, 108, and 116 of the prM protein in viral replication, we first generated a panel of JEV mutants, including three single-point mutants (rGI-K107R, rGI-K108R, and rGI-K116R), three double-point mutants (rGI-K107/108R, rGI-K107/116R, and rGI-K108/116R), and one triple-point mutant (rGI-K107/108/116R) using a reverse genetics system of the JEV Beijing/2020–1 strain [6] (Fig 3A). All mutants were successfully rescued, as confirmed by the robust NS1’ expression. All mutants remain genetically stable after five serial passages in BHK-21 cells, with no reversion mutations at the prM -K107R, -K108R, or -K116R sites (Fig 3B). Using these mutants, we further assessed the prM ubiquitination levels in BHK-21, ST, and DEF cells during JEV infection. The prM ubiquitination was reduced by single or double mutations and nearly abolished by the triple mutation (Fig 3C), which is consistent with the results generated with the ecotopic expression system (Fig 2), strongly suggesting that K107, K108, and K116 are critical ubiquitination sites on JEV prM. Further, the effect of impaired prM ubiquitination on viral growth kinetics was evaluated across multiple host cells. Although single-point mutants (rGI-K107R, rGI-K108R, and rGI-K116R) and double-point mutants (rGI-K107/108R, rGI-K107/116R, and rGI-K108/116R) exhibited comparable growth kinetics as the WT rGI in various cells, the triple-point mutant rGI-K107/108/116R with the deficiency of prM ubiquitination showed significantly reduced viral yields, with titers decreasing by 2.1- to 34.4-fold in BHK-21 cells, 4.1- to 14.6-fold in DEF cells, and 3.8- to 19.8-fold in ST cells from 12 to 60 hpi (Fig 3D). Notably, this replication defect was restricted to vertebrate cells, but not in C6/36 cells (Fig 3D). Therefore, the ubiquitination of prM is critical for efficient viral replication in vertebrate cells, but not in mosquito cells.

Orthoflavivirus prM proteins have been implicated in neurotoxicity [46,47]. We therefore evaluated the impact of prM ubiquitination on JEV virulence through a weanling mice challenge study. To respectively measure viral neurovirulence and neuroinvasiveness, mice (n = 5 per group) were intracerebrally (i.c.) inoculated with WT rGI or rGI-K107/108/116R at doses of 100, 500, and 1000 TCID_50_, or intraperitoneally (i.p.) inoculated with these two viruses at doses of 10^3^ and 10^5^ TCID_50_. Survival curves revealed that mice intracerebrally (i.c.) infected with the rGI or rGI-K107/108/116R strain had mortality rates of 100% at doses of 500 and 1000 TCID_50_, and mortality rates of 60% and 40% at doses of 100 TCID_50,_ respectively (Fig 3E). Meanwhile, no reversion of the prM-K107/108/116R mutation was observed in the brain samples of mice infected with rGI-K107/108/116R, indicating that rGI-K107/108/116R exhibits similar neurovirulence to WT rGI in mice. In contrast, intraperitoneal infection with rGI at doses of 10^5^ and 10^3^ TCID_50_ resulted in mouse death, with mortalities of 100% and 80% that were significantly higher (*p* = 0.0372; *p* = 0.0425) than those of 80% and 40% in mice intraperitoneal infected with rGI-K107/108/116R (Fig 3F). In addition, the survival times of mice infected with rGI were significantly shorter than those of mice infected with rGI-K107/108/116R (Fig 3F), and the histopathology examination further showed that mice infected with rGI exhibited more severe neuroinflammation, characterized by perivascular cuffing and meningitis (Fig 3G). Thus, the mutant rGI-K107/108/116R has attenuated neuroinvasiveness compared to the WT rGI.

In summary, our data showed that the ubiquitination of the JEV prM protein is critical for efficient viral replication in vertebrate cells and contributes to its neuroinvasiveness.

### The ubiquitination of prM confers differential adaptation of JEV in vertebrates and the mosquito vector

**Fig 4:**
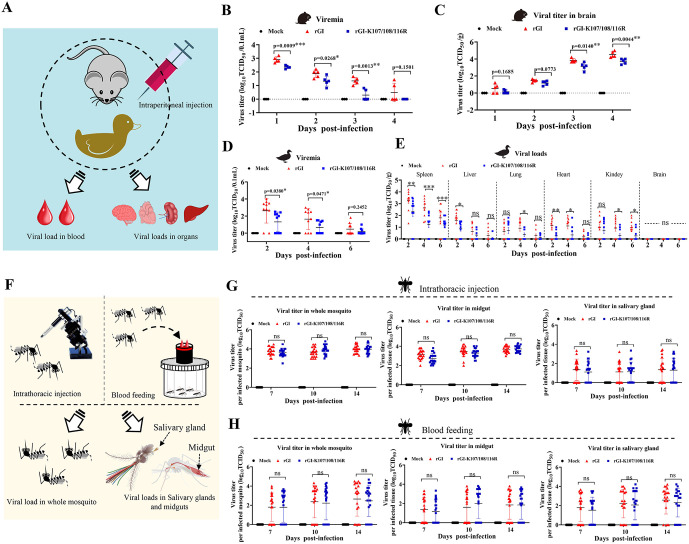
The ubiquitination of prM confers differential adaptation of JEV in vertebrate hosts and mosquito vectors. **(A)** The JEV infection models in the mouse and duckling. **(B, C)** Characterization of WT JEV and the K107/108/116R mutant in mice. Three-week-old weanling female C57BL/6 mice (n = 5) were intraperitoneally inoculated with 10^5^ TCID_50_ of rGI or rGI-K107/108/116R. Blood samples were collected through the submandibular vein for viremia analysis **(B)**. For the detection of viral loads in tissues, mice were euthanized at the indicated time points for tissue collection. Viral loads in tissues were titrated with TCID_50_ assays on BHK-21 cells **(C)**. **(D, E)** Characterization of WT JEV and the K107/108/116R mutant in ducklings. The two-day-old domestic ducklings (n = 10) were intraperitoneally inoculated with 10^5^ TCID_50_ of rGI or rGI-K107/108/116R. Blood samples were collected through the jugular vein for measurement of viremia in ducklings **(D)**. For the detection of viral loads in tissues. Ducklings per group were euthanized at the indicated time points for tissues collection. Viral loads in tissues were titrated with TCID_50_ assays on BHK-21 cells **(E)**. **(F)** A flow chart for the detection of JEV infection in whole mosquitoes and different tissues of *Aedes aegypti* mosquitoe*s* through blood feeding infection or intrathoracic inoculation. **(G, H)** Characterization of WT JEV and the K107/108/116R mutant in *Aedes aegypti* mosquitoe*s*. Viral loads in the whole mosquitoes, midgut, and salivary gland saliva were tested by TCID_50_ assays at 7, 10, and 14 dpi. Data are shown as means ± SD (B-E, G and **H)**. Statistical analysis was performed using the unpaired Student’s *t*-test. *p < 0.05, **p < 0.01, ***p < 0.001, ns, no significance.

JEV has a broad spectrum of hosts, encompassing mammals (pigs, humans, horses) [5,13], birds (migratory birds, waterfowl) [11,14], and the mosquito vector [48]. Given ubiquitination of orthoflavivirus proteins as a key determinant of viral host adaptation and transmission [41,42], we investigated the specific contribution of prM ubiquitination to host adaptation of JEV, using the established JEV infection models: C57BL/6 mice (mammal), domestic ducklings (avian), and *Aedes aegypti* mosquitoes. First, we intraperitoneally inoculated groups of mice (n = 5) with 10^5^ TCID_50_ of either the parental rGI or the mutant rGI-K107/108/116R, and monitored viral titers in blood and brain samples (Fig 4A). The JEV viremia was detectable as early as 1 dpi, with a viremic rate of 100% (5/5) both in rGI- and rGI-K107/108/116R- inoculated mice (Fig 4B). The viremia peaked at 1 dpi, but gradually declined over the next few days. The viremia in rGI-K107/108/116R-inoculated mice disappeared at 4 dpi, whereas a fraction of mice (2/5) inoculated with rGI remained viremic (Fig 4B). In addition, the parental rGI-inoculated mice produced 0.54 to 1.05 log higher viremia compared to the mice inoculated with the rGI-K107/108/116R at 1 (*p* = 0.0009), 2 (*p* = 0.0268), and 3 dpi (*p* = 0.0013) (Fig 4B). Consistent with this, the viral loads in the brains of rGI-inoculated mice were significantly higher than those in rGI-K107/108/116R-inoculated mice at 3 and 4 dpi (Fig 4C), demonstrating that the K107/108/116R mutations attenuate viral replication efficiency in mice.

JEV-infected ducks could produce sufficient viremia for mosquito infection [11]. We subsequently monitored the levels of viremia and tissue tropism in JEV-inoculated ducklings (Fig 4A). The mutant virus exhibited attenuated replication in ducklings, evidenced by a lower viremic rate and significantly reduced viremia observed in rGI-K107/108/116R-infected ducklings at 2 (p = 0.0380) and 4 dpi(p = 0.0471) (Fig 4D). Moreover, the viral loads in various tissues were assessed at different time points after infection. JEV was detected in the spleens, livers, lungs, hearts, and kidneys, but not in the brains, and higher viral loads were observed in the spleens (Fig 4E). The viral loads in the spleens of rGI-inoculated ducklings were significantly higher than those in rGI-K107/108/116R-inoculated ducklings at 2, 4, and 6 dpi (Fig 4E). Similar results were also observed in the livers, lungs, heart and kidneys, with lower viral titers in tissues of rGI-K107/108/116R-inoculated ducklings (Fig 4E). These *in vivo* results demonstrate that the K107/108/116R mutations attenuate JEV replication in ducklings, corroborating our *in vitro* findings (Fig 3D).

Having shown that prM protein ubiquitination does not affect JEV replication in mosquito cells (Fig 3D). To obtain more evidence to support this result, we therefore further evaluated the replication ability of rGI and rGI-K107/108/116R in mosquitoes. *Aedes aegypti* mosquitoes were exposed to rGI-K107/108/116R and WT through blood feeding with 10^6^ TCID_50_/mL viruses or intrathoracic (i.t.) injection with 500 TCID_50_ per mosquito (Fig 4F). Viral titers in whole mosquito (n = 20), midgut (n = 20), and salivary gland (n = 20) at 7, 10, and 14 dpi were measured (Fig 4F). No statistical difference in the infection rates between GI- and rGI-K107/108/116R- infected mosquitoes was observed at 7 dpi, 10dpi, and 14 dpi, with infection rates of 60% to 75% for mosquitoes infected via blood feeding (Fig 4G), and 100% for mosquitoes infected via i.t. injection (Fig 4H). Additionally, among JEV-positive mosquitoes, comparable viral titers between rGI and rGI-K107/108/116R were observed in whole mosquitoes, midgut, and salivary gland (Fig 4G and 4H). The results showed that the deficiency of prM ubiquitination has no effect on JEV replication in mosquitoes.

Therefore, these results showed that the ubiquitination of prM contributes to the adaptation of JEV in vertebrate hosts, but not in the mosquito vector.

### Lysines at 107, 108, and 116 of prM serve as conserved ubiquitination sites across multiple mosquito-borne orthoflaviviruses

**Fig 5:**
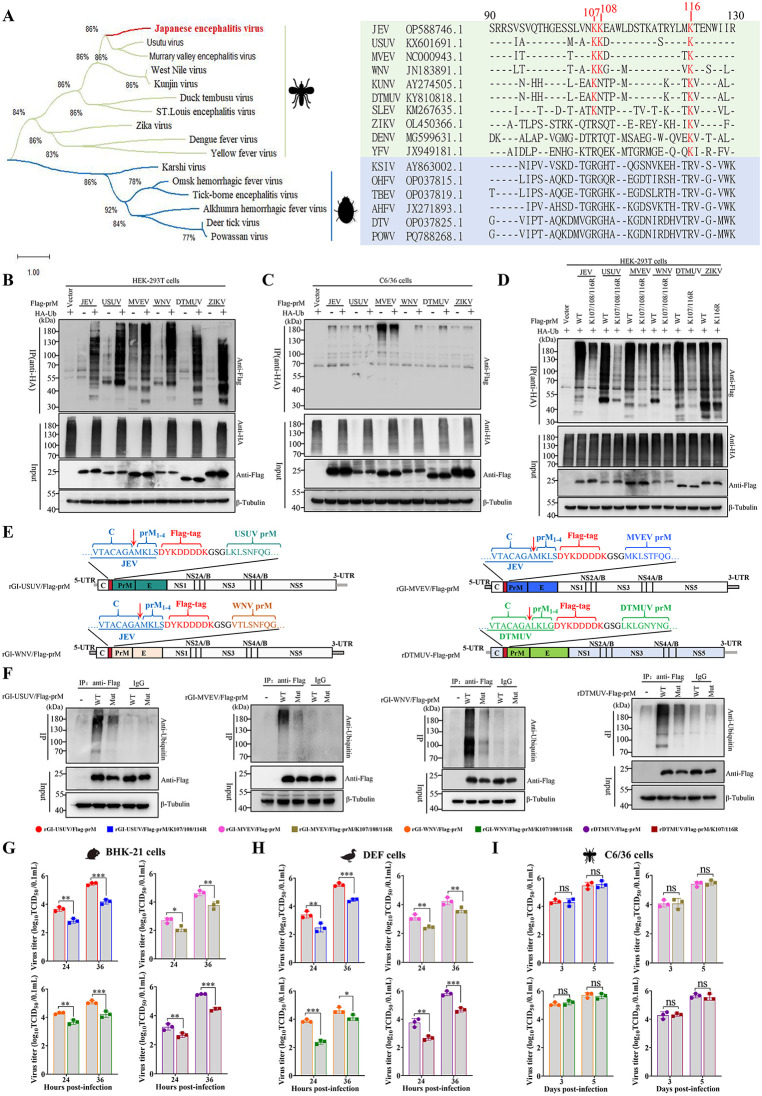
Lysines at 107, 108, and 116 of prM serve as conserved ubiquitination sites across multiple mosquito-borne orthoflaviviruses. **(A)** Phylogenetic analysis and multiple sequence alignments of the genus *Orthoflavivirus* were performed with the MEGA version 12 and DNASTAR software, respectively. The number highlighted in red indicates the lysine residues at 107, 108, and 116 of the prM proteins. **(B-D)** Ubiquitination analysis of the prM proteins of mosquito-borne orthoflaviviruses. HEK-293T (B, D) and C6/36 (C) cells were respectively transfected with a plasmid expressing a Flag-tagged prM or the corresponding mutants, along with a plasmid expressing HA-Ub or an empty vector. At 36 hpt, cells were harvested for immunoprecipitation assays using anti-HA magnetic beads. The ubiquitination of the prM proteins was analyzed by immunoblotting with an anti-Flag antibody. **(E)** Genomic structures of the chimeric viruses, rGI-USUV/Flag-prM, rGI-WNV/Flag-prM, rGI-MVEV/Flag-prM, and rDTMUV-Flag-prM. The arrow indicates the cleavage site between the C and prM protein. The chimeric viruses rGI-USUV/Flag-prM, rGI-WNV/Flag-prM, and rGI-MVEV/Flag-prM were created by replacing the coding region of JEV structural proteins (C, prM, and E) with the corresponding sequences from USUV, WNV, or MVEV. The recombinant virus rDTMUV-Flag-prM was constructed using a PCR-based reverse genetics system based on the whole DTMUV genome. **(F)** The ubiquitination of Flag-prM in BHK-21 cells infected with rGI-USUV/Flag-prM, rGI-WNV/Flag-prM, rGI-MVEV/Flag-prM, rDTMUV-Flag-prM, or their corresponding mutants. **(G-I)** The growth kinetics of chimeric viruses or their mutants in BHK-21 cells **(G)**, DEF (H) and C6/36 cells **(I)**. Data are shown as means ± SD (G to **I)**. Statistical analysis was performed using the unpaired Student’s *t*-test. *p < 0.05, **p < 0.01, ***p < 0.001, ns, no significance.

The prM protein exhibits functional conservation in orthoflaviviruses. To explore whether prM ubiquitination exerts a regulatory effect on the host adaptation of other arthropod-borne orthoflavivirus, we performed multiple sequence alignments to evaluate the conservation of residues K107, K108, and K116 on prM across arthropod-borne orthoflavivirus. Remarkably, the lysine residues at positions 107, 108, or 116 of the prM protein are strictly unique to mosquito-borne orthoflavivirus, but not to tick-borne orthoflaviviruses, with K116 completely conserved across mosquito-borne orthoflaviviruses (Fig 5A). Importantly, the residues K107, K108, and K116 of prM are highly conserved in a specific clade of mosquito-borne orthoflaviviruses closely related to JEV, including USUV, MVEV and WNV (Fig 5A). Thus, we propose that the ubiquitination sites K107, K108, and K116 on the prM protein are evolutionarily selected in mosquito-borne orthoflavivirus, and may serve as a critical determinant for their adaptation to vertebrate hosts. To validate this speculation, a panel of plasmids expressing Flag-prM from USUV, MVEV, WNV, DTMUV, and ZIKV, as well as their corresponding mutants, was constructed and subjected to ubiquitination analysis. As expected, the WT prM proteins from USUV, MVEV, WNV, DTMUV, and ZIKV underwent strong ubiquitination in HEK-293T cells (Fig 5B), but not in mosquito cells (Fig 5C). Notably, the arginine substitutions at these conserved lysines significantly reduced the ubiquitination of USUV, MVEV, WNV, and DTMUV prM proteins (Fig 5D). In contrast, the ZIKV prM K116R mutant retained significant ubiquitination, indicating that K107, K108, and K116 are the key ubiquitination sites for USUV, MVEV, WNV, and DTMUV prM, whereas ZIKV prM possesses additional ubiquitination sites beyond K116. To further confirm the critical role of these conserved lysines in prM ubiquitination, we created a panel of chimeric viruses (rGI-USUV/Flag-prM, rGI-WNV/Flag-prM, rGI-MVEV/Flag-prM) by replacing the coding region of JEV structural proteins (C, prM, and E) with the corresponding sequences from USUV, WNV, or MVEV (Fig 5D), respectively. Additionally, a PCR-based reverse genetics system based on the whole DTMUV genome was utilized to construct recombinant virus rDTMUV-Flag-prM[49].To overcome the lack of specific antibodies against prM of these orthoflaviviruses, a Flag-tag was inserted into the junction between C and prM genes (Fig 5E). Immunofluorescence assay confirmed that Flag-positive signals were detected only in cells infected with Flag-prM chimeric viruses (S3A and S3B Fig). Furthermore, all chimeric viruses remained genetically stable over five serial passages in BHK-21 cells, with no mutations or deletions observed in the Flag-tag sequence (S3C Fig). We introduced triple mutation K107/108/116R into the rGI-USUV/Flag-prM, rGI-WNV/Flag-prM, and rGI-MVEV/Flag-prM, or double mutation K107/116R into the rDTMUV-Flag-prM (S3A Fig). As expected, the polyubiquitination of WT prM was almost completely abolished by the mutations introduced at K107, K108, and K116 residues (Fig 5F), confirming that these lysines serve as conserved ubiquitination sites in the four mosquito-borne orthoflaviviruses, including USUV, WNV, MVEV, and DTMUV.

Further, the growth kinetics of chimeric viruses with impaired prM ubiquitination were assessed in multiple host cells including BHK-21, DEF, and C6/36 cells. All mutant chimeric viruses exhibited comparable replication kinetics to that of WT chimeric viruses in C6/36 cells (Fig 5I), while the yields of chimeric viruses in DEF and BHK-21 cells were significantly reduced compared to the WT chimeric viruses (Fig 5G and 5H). Therefore, these results further supported the evolutionarily conserved role of prM ubiquitination in facilitating the adaptation of multiple mosquito-borne orthoflavivirus, including JEV, USUV, WNV, MVEV, and DTMUV, to vertebrate hosts.

### The ubiquitination of JEV prM protein is involved in viral assembly in vertebrate cells

**Fig 6:**
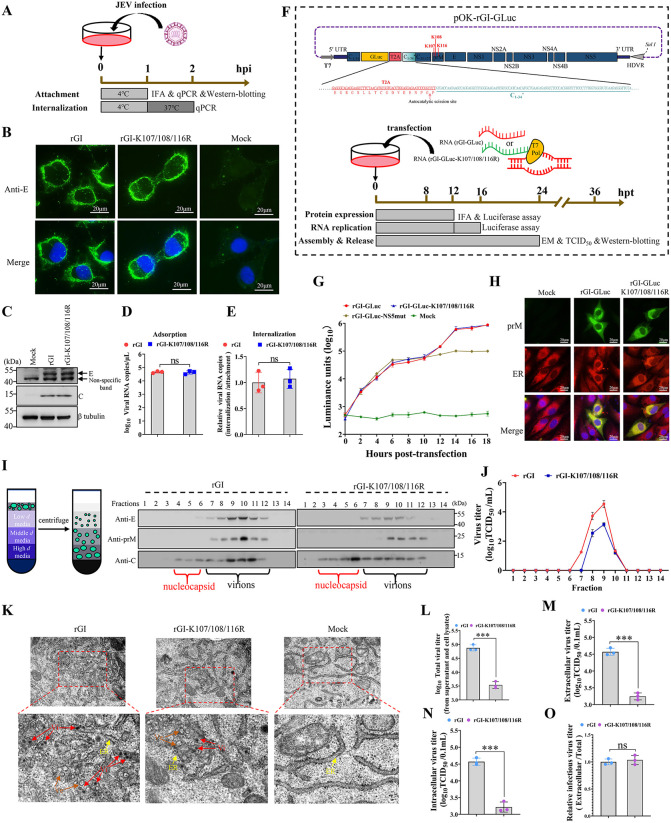
The ubiquitination of JEV prM protein is involved in viral assembly in target cells. **(A)** Schematic diagram of viral attachment and internalization assays. **(B-D)** Attachment assay. BHK-21 cells were incubated with JEV at an MOI of 10 for 1 hour at 4°C to allow adsorption. IFA **(B)**, Western blotting analysis **(C)**, and RT-qPCR (D) were performed to quantify the attached viral particles. **(E)** Internalization assay. BHK-21 cells were infected with JEV at an MOI of 10 for 1 hours, and then shifted to 37°C for 1 h. RT-qPCR was conducted to quantify the efficiency of internalization. **(F)** Experiment design for examining viral protein expression, RNA synthesis, and release using recombinant viruses expressing GLuc. **(G)** BHK-21 cells were transfected with equal doses of rGI-GLuc or rGI-GLuc-K107/108/116R RNA. At the indicated time points, culture supernatants were collected to determine luciferase activities. In this system, initial translation of viral RNA occurs from 0 to 6 hours post‑transfection, and GLuc accumulation during this first round directly reflects viral RNA translation levels. Subsequently, from 6 to 10 hours post‑transfection, the first round of replication takes place, after which newly synthesized viral RNA drives a second round of translation starting at 10 hpt. GLuc accumulation in this second round thus represents the level of RNA replication. **(H)** Immunofluorescence analysis of BHK-21 cells transfected with the equal doses of rGI-GLuc or rGI-GLuc-K107/108/116R RNA using anti-prM (green) or anti-calnexin (red) antibodies. Scale bar: 20 μm. **(I, J)** Sucrose density-gradient analysis of intracellular viral particles derived from BHK-21 cells transfection with equal doses of rGI or rGI-K107/108/116R RNA. Fourteen fractions were collected and subjected to measurement of the nucleocapsid and intracellular particle by immunoblotting (I) and TCID_50_ assays **(J)**. **(K)** Electron microscopy analysis of BHK-21 cells transfected with rGI or rGI-K107/108/116R RNA. ER, virions (Vi), and vesicles (Ve) are indicated by the yellow arrow, red arrow, and orange arrow, respectively. **(L)** The total viral titer from supernatant and cell lysates in BHK-21 cells. **(M, N)** The extracellular (M) and intracellular virus titers (N) in BHK-21 cells. **(O)** Comparison of extracellular and total infectious viral titers. Data are shown as means ± SD (D, E, G, J, L, M, N and **O)**. Statistical analysis was performed using the unpaired Student’s *t*-test. ***p < 0.001, ns, no significance.

To elucidate the mechanisms by which prM ubiquitination contributes to the adaptation of JEV in vertebrate hosts, we systematically evaluated the effect of K107/108/116R triple mutation on various stages of the JEV replication cycle, including attachment, internalization, viral translation, RNA replication, virion assembly, and release in BHK-21 and ST cells (Fig 6). To specifically assess whether K107, K108, and K116 are involved in viral attachment and internalization, BHK-21 and ST cells were incubated with rGI or K107/108/116R mutant at an MOI of 10 for 1 hour at 4°C to allow adsorption, followed by a temperature shift to 37 °C for 1 h to permit internalization (Fig 6A). The K107/108/116R triple mutation did not impair either viral attachment or internalization in BHK-21 and ST cells, as evidenced by the comparable levels of structural proteins detected via IFA and Western blot analysis during adsorption (Figs 6B, 6C, S4A and S4B), as well as the similar viral RNA levels measured by RT-qPCR both in adsorption and internalization (Figs 6D, 6E, S4C and S4D). To dissect the impact of prM ubiquitination on viral translation and replication stages, a recombinant GLuc reporter system (rGI-GLuc; Fig 6F) was used to distinguish viral translation from RNA synthesis, with a replication-defective NS5 mutant (rGI-GLuc-NS5mut) as a replication-deficiency control. In this system, viral translation could be determined based on the accumulation of GLuc before 10 hpt, whereas viral RNA synthesis could be assessed based on the second round of GLuc accumulation. In BHK-21 or ST cells transfected with equal doses of viral RNA, the rGI-GLuc-K107/108/116R exhibited comparable translation and replication efficiency to rGI-GLuc (Figs 6G and S4E), as measured by luciferase assays from 2 to 18 hpt. Given the ubiquitination involved in protein intracellular localization [49], we also analyzed the impact of ubiquitination deficiency on the subcellular localization of prM. The K107/108/116R triple mutation did not alter the subcellular localization of the prM protein in the endoplasmic reticulum in BHK-21 cells (Fig 6H). Collectively, these results demonstrate that the loss of prM ubiquitination does not affect viral attachment, internalization, protein expression, and RNA replication.

Previous studies have established that the prM protein is essential for virion assembly and release in the genus *Orthoflavivirus* [19]. We further characterized the density of intracellular virus particles via isopycnic separation to determine whether prM ubiquitination is required for viral assembly. BHK-21 and ST cells transfected with the equivalent doses of WT or mutated viral RNA were lysed, and then layered on top of a sucrose gradient (10%-60%) and centrifuged for 3 hours (Fig 6I). Fourteen fractions were collected and subjected to measurement of the nucleocapsid and intracellular particle by immunoblotting, and infectivity by TCID_50_ assays. For both viruses, a prominent peak of virion was observed in fractions 8–10, which coincided with the peak of infectivity both in BHK-21 and ST cells (Figs 6I, 6J and S4F). Of note, the deficiency of prM ubiquitination suppresses the assembly of viral particles and increases the accumulation of non-enveloped nucleocapsids in fractions 3–6 of the gradient (Figs 6I, S4F and S4G). The effects of prM protein ubiquitination defect on viral particle assembly in BHK-21 cells were further investigated by negative-staining electron microscopy. As shown in Fig 6K and 6I, the deficiency of prM ubiquitination did not completely abolish viral particle assembly but significantly reduced total viral titers (intracellular and extracellular) compared to the WT virus. Consistent with this, a similar viral titer reduction was also observed in ST cells (S4H Fig), suggesting that the ubiquitination of the JEV prM protein facilitates viral assembly. Furthermore, comparable release efficiencies between WT and mutant indicated that ubiquitination of prM protein did not affect viral release both in BHK-21 and ST cells (Fig 6M-6O, and S4I-4K).

In summary, these results showed that the ubiquitination of the JEV prM protein is mainly involved in viral assembly in vertebrate cells.

### The ESCRT proteins mediate JEV assembly driven by prM ubiquitination

**Fig 7:**
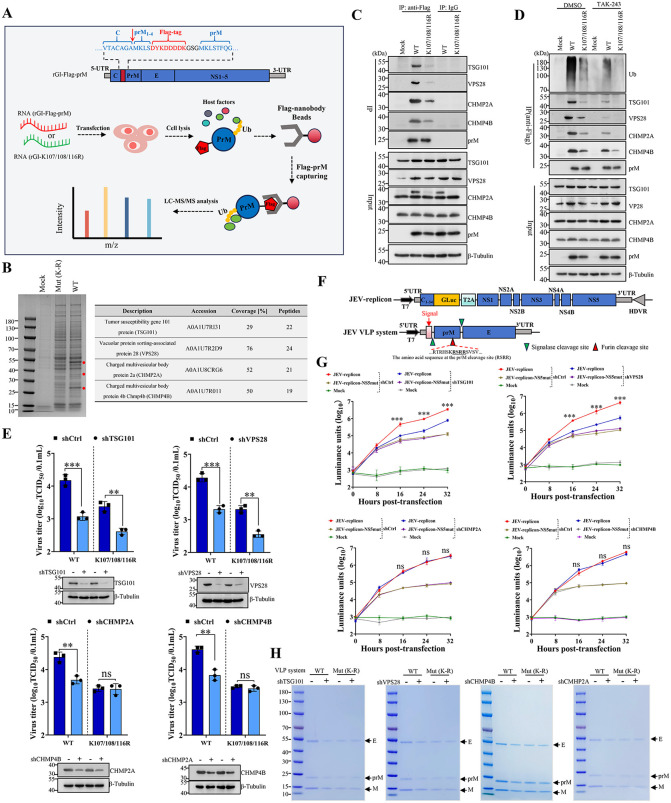
The ubiquitination of prM facilitates efficient JEV assembly by recruiting ESCRT proteins TSG101, VPS28, CHMP2A, and CHMP4B. **(A)** A workflow for the identification of prM-interacting host factors through mass spectrometry analysis using a recombinant virus rGI-Flag-prM. BHK-21 cells transfected with equal viral RNA (3µg) from rGI-Flag-prM or its ubiquitination-deficient mutant (K107/108/116R) were immunoprecipitated at 12 hpt with Flag-nanobody magnetic beads. The immunoprecipitates were separated by SDS-PAGE, stained with Coomassie Blue, and the ubiquitinated prM-associated bands were excised, trypsin-digested, and analyzed by LC-MS/MS. **(B)** Identification of cellular proteins associated with ubiquitinated prM protein. The specific or enriched protein bands associated with ubiquitinated prM protein in BHK-21 cells were detected by SDS-PAGE and identified by mass spectrometry. The red dots indicate the protein bands that were excised for mass spectrometry analysis. **(C)** The interaction between JEV prM and ESCRT proteins was validated by co-immunoprecipitation analysis. BHK-21 were either mock-infected or infected with rGI-Flag-prM or rGI-Flag-prM-K107/108/116R at an MOI of 1. At 24 hpi, cells were lysed for immunoprecipitation with an anti-Flag nanobody. The prM protein and cellular proteins are detected by Western blot analysis. **(D)** Immunoprecipitation analysis of prM protein ubiquitination and its interacting cellular proteins in BHK-21 cells infected with rGI-Flag-prM or rGI-Flag-prM-K107/108/116R in the presence or absence of TAK243. **(E)** The effect of gene silencing of ESCRT proteins on JEV infection. BHK-21 cells with the knock-down expression of TSG101, VPS28, CHMP2A or CHMP4B were infected with rGI and rGI-prM-K107/108/116R at an MOI of 0.5. At 24 hpi, viral titers in culture supernatants were measured by TCID_50_ assays. **(F)** The schematic diagrams of the JEV replicon and VLP system. **(G)** BHK-21 cells with the knock-down of TSG101, VPS28, CHMP2A or CHMP4B were transfected with equal doses of RNA derived from JEV replicon or JEV replicon-NS5mut. At the indicated time points of post-transfection, culture supernatants were collected to determine luciferase activities. **(H)** BHK-21 cells with the knock-down of TSG101, VPS28, CHMP2A or CHMP4B were transfected with RNA derived from the JEV VLP system. At 36 hpt, VLPs in the cell supernatant were collected, concentrated, and purified, followed by quantification via SDS-PAGE. Data are shown as means ± SD **(E, G)**. Statistical analysis was performed using the unpaired Student’s *t*-test. *p < 0.05, **p < 0.01, ***p < 0.001, ns, no significance.

Viral assembly necessitates the coordination of viral proteins with multiple host factors. To elucidate the specific molecular mechanism of prM protein ubiquitination regulating the assembly of JEV, cellular proteins that associate with ubiquitinated prM protein were identified using the immunoprecipitation assay followed by LC-MS/MS analysis (Fig 7A). Recombinant viruses with a Flag-tag at the N-terminus of prM were rescued for the immunoprecipitation assay and designated as rGI-Flag-prM and rGI-Flag-prM-K107/108/116R (Figs 7A and S5A). Two recombinant viruses exhibited replication kinetics comparable to their parental viruses without Flag-tag insertion (S5B Fig), and maintained genetically stable over five serial passages in BHK-21 cells (S5C Fig). In the Flag-tag immunoprecipitated protein complex, three unique or enriched bands specifically associated with the ubiquitinated prM were selected for LC-MS/MS analysis (Fig 7B). Among the identified proteins, ESCRT components (TSG101, VPS28, CHMP2A, and CHMP4B) showed relatively high abundance and significant enrichment (S2 Table and S6 Fig). Notably, several ESCRT associated proteins including TSG101, CHMP2A, and CHMP4B are also required for orthoflavivirus particle formation on the ER Membrane [30]. Therefore, we selected ESCRT as a candidate host factor for further investigation. To further confirm the recruitment of these ESCRT proteins depends on prM ubiquitination, we evaluated the interactions between prM and these ESCRT proteins in BHK-21 cells infected with rGI-Flag-prM or the ubiquitination-deficient mutant by co-immunoprecipitation assays. As expected, ESCRT proteins TSG101, VPS28, CHMP2A, and CHMP4B were recruited to the ubiquitinated prM protein, whereas the recruitment of these ESCRT proteins was dramatically impaired for the ubiquitination-deficient prM mutant (Fig 7C). In addition, the disruption of prM ubiquitination by TAK-243 treatment impaired the recruitment of these ESCRT proteins to prM (Fig 7D). These results indicate that prM ubiquitination is critical for its interactions with the ESCRT proteins.

The role of TSG101, VPS28, CHMP2A, and CHMP4B proteins in JEV infection was further evaluated. Knockdown of each protein via shRNA dramatically attenuated rGI replication, with titers reduced by approximately 5.0- to 12.9-fold (Fig 7E), confirming their involvement in the JEV life cycle. Notably, the depletion of TSG101 or VPS28 attenuated the replication of both WT rGI and the ubiquitination-deficient mutant, whereas knockdown of CHMP2A or CHMP4B specifically inhibited the replication of WT virus but not the mutant (Fig 7E), indicating that CHMP2A and CHMP4B regulate JEV replication strictly dependent on prM ubiquitination. Considering that the important roles of ESCRT in viral RNA replication and assembly of enveloped viruses [22], we further sought to pinpoint the specific stage at which TSG101, VPS28, CHMP2A, and CHMP4B contribute to the JEV propagation. To this end, we employed a JEV replicon system to assess viral RNA replication and a virus-like particle (VLP) assay to examine viral assembly (Fig 7F). Based on GLuc activities from 8 to 36 hpt, the silence of TSG101 and VPS28 significantly attenuated viral RNA synthesis, whereas the knockdown of CHMP2A and CHMP4B had no detectable effect on this process, revealing the involvement of TSG101 and VPS28 in JEV RNA replication (Fig 7G). In contrast, all four ESCRT proteins were required for efficient viral assembly. The knockdown of TSG101, VPS28, CHMP2A, and CHMP4B significantly attenuated viral assembly, with lower levels of VLPs observed in knock-down cells transfected with WT VLP systems (Fig 7H); however, this attenuation was abolished when using a VLP system carrying the ubiquitination-deficient prM mutant (Fig 7H), suggesting that ESCRT components (TSG101, VPS28, CHMP2A, CHMP4B) mediate JEV assembly in a manner strictly dependent on prM ubiquitination.

Together, these results demonstrate that TSG101, VPS28, CHMP2A, and CHMP4B are recruited in a prM ubiquitination-dependent manner to facilitate JEV assembly.

### TSG101 acts as an adaptor bridging ubiquitylated prM and downstream ESCRTs to promote viral budding in vertebrate hosts, but not in mosquitoes

**Fig 8:**
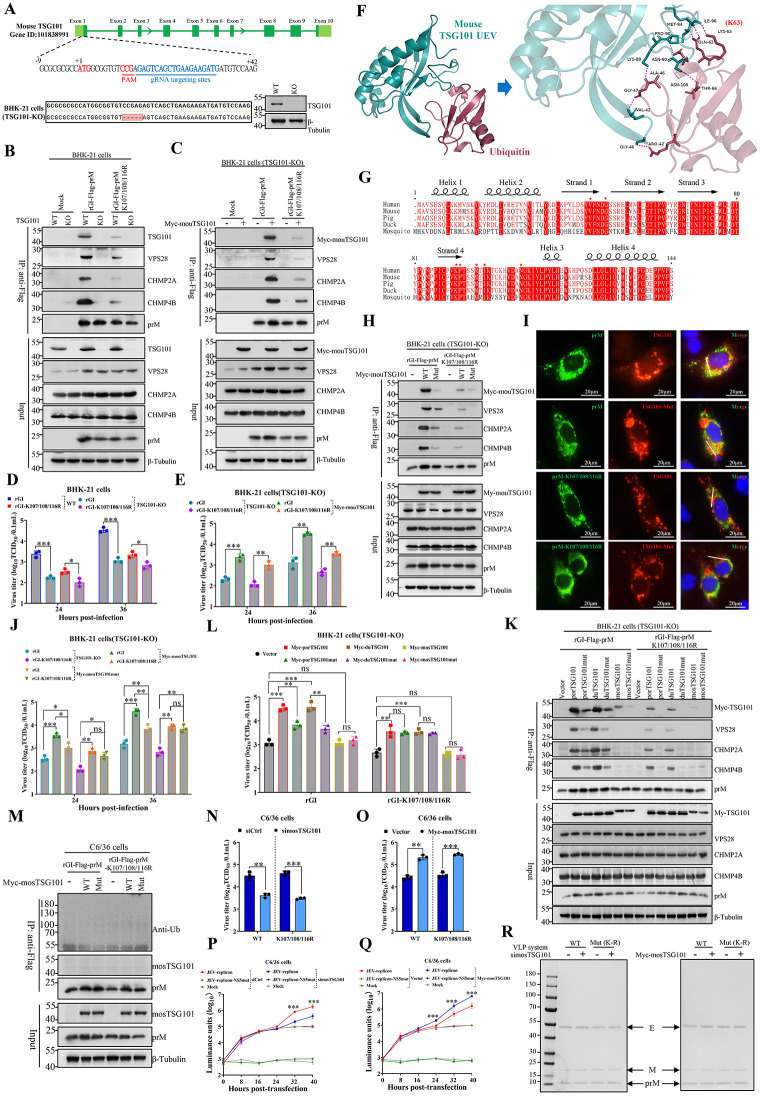
TSG101 acts as an adaptor bridging ubiquitylated prM and downstream ESCRT proteins to promote viral budding in vertebrate hosts, but not in mosquitoes. **(A)** Sequence analysis of the *TSG101* gene in WT and TSG101-KO BHK-21 cells. TSG101 expression in WT and TSG101-KO BHK-21cell lines was validated by Western blotting. **(B)** The interaction between prM and ESCRT proteins in JEV-infected BHK-21 cells. Cells were either mock-infected or infected with rGI-Flag-prM or rGI-Flag-prM-K107/108/116R at an MOI of 1. At 24 hpi, cells were lysed for immunoprecipitation with anti-Flag nanobody-conjugated magnetic beads. The immunoprecipitated prM protein and cellular proteins were analyzed by Western blotting. **(C)** Immunoprecipitation analysis of prM protein and its interacting cellular proteins in TSG101-KO BHK-21 cells infected with rGI-Flag-prM or rGI-Flag-prM-K107/108/116R in the presence or absence of Myc-mosTSG101 over-expression. **(D)** The growth kinetics of rGI and rGI- prM-K107/108/116R in WT and TSG101-KO BHK-21 cells. **(E)** The growth kinetics of rGI and rGI- prM-K107/108/116R in TSG101-KO BHK-21 cells with the over-expression of mosTSG101. **(F)** The interaction interface between the TSG101 UEV domain and Ub. **(G)** Amino acid sequences and secondary structures of TSG101 UEV domain. Conserved residues are shaded red. TSG101 UEV residues involved in binding Ub are indicated by red dots. **(H)** Immunoprecipitation analysis of prM protein and its interacting cellular proteins in TSG101-KO BHK-21 cells infected with rGI-Flag-prM or rGI-Flag-prM-K107/108/116R with overexpression of Myc-mosTSG101 or Myc-TSG101-Mut. **(I)** Co-localization analysis between prM or prM-K107/108/116R, and Myc-mouTSG101 or Myc-mouTSG101-Mut in TSG101-KO BHK-21 cells. The linear region of the co-localization analysis was labeled with a white line (related to S8 Fig). **(J)** The growthkinetics of rGI and rGI-prM-K107/108/116R in TSG101-KO BHK-21 cells with the overexpression of Myc-mouTSG101 or Myc-mouTSG101-Mut. **(K)** Immunoprecipitation analysis of prM protein and its interacting cellular proteins in TSG101-KO BHK-21 cells infected with rGI-Flag-prM or rGI-Flag-prM-K107/108/116R in the presence or absence of the over-expression of Myc-porTSG101, Myc-duTSG101, or Myc-mosTSG101. **(L)** The growth kinetics of rGI and rGI-prM-K107/108/116R in TSG101-KO BHK-21 cells with the expression of Myc-TSG101 derived from pig (porTSG101), duck (duTSG101), and mosquito (mosTSG101), or their corresponding mutants. **(M)** Immunoprecipitation analysis of prM protein ubiquitination and Myc-mosTSG101 in C6/36 cells infected with rGI-Flag-prM or rGI-Flag-prM-K107/108/116R in the presence or absence of the over-expression of Myc-mosTSG101 or Myc-mosTSG01-Mut. **(N, O)** The growth kinetics of rGI and rGI- prM-K107/108/116R in C6/36 cells with mosTSG101 knockout (N) or overexpression **(O)**. **(P, Q)** The luciferase activities of C6/36 cells transfected with JEV replicon RNA in the presence of shmosTSG101 treatment (P) or Myc-mosTSG01 overexpression **(Q)**. [*] indicated the significant difference in JEV replicon between the shCtrl group and shTSG101 group, as well as the vector group and Myc-mosTSG101 group. **(R)** The quantification of JEV VLPs in the C6/36 cells in the presence of shmosTSG101 treatment or Myc-mosTSG01 over-expression. Data are shown as means ± SD (D-E, G, L, and N-Q). Statistical analysis was performed using the unpaired Student’s *t*-test. *p < 0.05, **p < 0.01, ***p < 0.001, ns, no significance.

Our findings identified TSG101, VPS28, CHMP2A, and CHMP4B as important cofactors for JEV particle assembly recruited by ubiquitylated prM (Fig 7). Given the established role of TSG101 as the most upstream ESCRT factor [50] for recruiting the entire ESCRT machinery, we further investigated its potential role as an adaptor bridging ubiquitylated prM and downstream ESCRT components. To this end, we generated a TSG101-KO BHK-21 cell line by introducing a five-nucleotide deletion in exon 1 of the TSG101 gene, which completely abolished TSG101 expression without affecting cell proliferation (Fig 8A). As shown in Fig 8B, TSG101 deficiency abrogated the recruitment of the downstream ESCRT components VPS28, CHMP2A, and CHMP4B by ubiquitinated prM. However, this defect was effectively rescued through the ectopic expression of mouse-derived Myc-tagged TSG101 (Myc-mouTSG101), as evidenced by the restored formation of the TSG101/VPS28/CHMP2A/CHMP4B complex in the knockout cells (Fig 8C). Additionally, overexpression of TSG101 can directly promote the recruitment of downstream ESCRT components VPS28, CHMP2A, and CHMP4B by ubiquitinated prM (S7A Fig), while the knockdown of VPS28, CHMP2A, and CHMP4B had no detectable effect on the recruitment of TSG101 driven by prM protein (S7B-7D Fig), indicating that TSG101 functions as a critical adaptor bridging the interactions between ubiquitylated prM and downstream ESCRT components. The growth of rGI and rGI-K107/108/116R in TSG101-KO cells was significantly alleviated at 24 hpi and 36 hpi compared to that in WT cells (Fig 8D). This replication defect was rescued by the reintroduction of Myc-mouTSG101 in TSG101-KO cells (Fig 8E), further confirming the decisive role of TSG101 in JEV infection.

TSG101 plays important roles in recognizing ubiquitylated protein cargo and recruiting the downstream ESCRT complexes [50,51]. Similar to E2 ligases, TSG101 binds ubiquitin directly through its N-terminal UEV domain [52]. Analysis of the three-dimensional structure of TSG101 UEV-Ub complex revealed that UEV consists of four helices packed against one side of a four-stranded antiparallel β-sheet (Fig 8F and 8G), and mainly interacts with ubiquitin through intermolecular hydrogen bonds between Val_42_-Ub Gly_47_, Gly_46_ -Ub Arg_42_, Lys_89_ -Ub Ala_46_, Pro_90_ -Ub Asn_60_, Met_94_ -Ub Gln_62_, Ile_96_ -Ub Lys_63_, and Asn_105_ -Ub Thr_66_ (Fig 8F). To determine whether the UEV-Ub interaction is required for the ESCRT recruitment mediated by ubiquitylated prM, we introduce alanine (Ala) substitutions in the TSG101 UEV domain to disrupt the key hydrogen bonds between TSG101 UEV and ubiquitin. The alanine substitutions in TSG101 UEV domain significantly diminish its binding affinity for the ubiquitylated prM, thereby impairing the recruitment of downstream ESCRT components VPS28/CHMP2A/CHMP4B (Fig 8H). Consistent with this, a marked decrease in colocalization was observed between WT prM and TSG101-Mut, as well as between the ubiquitination-deficient prM mutant (prM-K107/108/116R) and WT TSG101 (Fig 8I), indicating that the recruitment of ESCRT by ubiquitylated prM requires specific UEV-Ub interaction. Furthermore, the growth kinetics analysis of the virus showed that the replication advantage conferred by WT TSG101 was specifically dependent on prM ubiquitination (Fig 8J). Although ectopic expression of either TSG101 or TSG101-Mut enhanced the replication of both rGI and rGI-K107/108/116R in TSG101-KO BHK-21 cells, rGI replicated more robustly in the presence of WT TSG101(Fig 8J). In contrast, the rGI-K107/108/116R mutant replicated to similar levels in the presence of either TSG101 or TSG101-Mut protein (Fig 8J and S8 Fig). These results indicate that the specific interaction between the TSG101 UEV domain and ubiquitinated prM plays a critical regulatory role in JEV replication, and TSG101 can also promote JEV infection in the absence of prM ubiquitination.

Given the high conservation of the ESCRT pathway across eukaryotes, we sought to determine whether the function of TSG101 in regulating JEV replication is conserved in various species. A comparison of TSG101 amino acid sequences from human, mouse, pig, duck, and mosquito revealed that the UEV domain is highly conserved among vertebrates but exhibits substantial divergence in mosquitoes (Fig 8G). Notably, the specific residues forming the ubiquitin-binding interface (Val_42_, Gly_46_, Lys_89_, Pro_90_, Met_94_, Ile_96_, and Asn_105_) showed complete conservation across various species, including mosquitoes (Fig 8G). To assess whether this sequence conservation translates to functional similarity, the recruitment of ESCRT components by prM was further evaluated by immunoprecipitation assay in TSG101-KO BHK-21 cells with the ectopic expression of pig-derived Myc-tagged TSG101 (Myc-porTSG101), duck-derived Myc-tagged TSG101 (Myc-duTSG101), mosquito-derived Myc-tagged TSG101 (Myc-mosTSG101), or their corresponding mutants. As shown in Fig 8K, WT prM protein efficiently recruited porTSG101 and duTSG101 to assemble the ESCRT complex in BHK-21 cells, which was disrupted by mutations at either prM ubiquitination sites or TSG101 ubiquitin-binding sites. Intriguingly, mosquito TSG101 (mosTSG101) was also recruited by ubiquitinated prM, but failed to recruit the downstream ESCRT machinery (Fig 8K). This functional deficit is attributed to its inability to interact with VPS28, CHMP2A, and CHMP4B in BHK-21 cells (S9A Fig). Meanwhile, the growth kinetics analysis showed the viral replication ability of rGI and rGI-K107/108/116R was significantly enhanced in TSG101-KO BHK-21 cells with the ectopic expression of either porTSG101 or duTSG101, but not mosTSG101(Fig 8L). Therefore, the function of TSG101 in regulating JEV replication is conserved in vertebrate hosts, but not in mosquitoes.

Although the JEV prM protein is not ubiquitinated in mosquito-derived C6/36 cells (Fig 1), mosquito-derived TSG101 can be recruited by ubiquitinated prM in heterologous BHK-21 cells (Fig 8K). To resolve this discrepancy and determine whether an interaction between prM and mosTSG101 nevertheless occurs in a native mosquito cell, we performed an immunoprecipitation assay in C6/36 cells with the over-expression of Myc-mosTSG101. As expected, no interaction was detected between prM and either WT or ubiquitin-binding-deficient mosTSG101 (mosTSG101-Mut) in C6/36 cells (Fig 8M). In addition, prM also failed to recruit the downstream components VPS28, CHMP2A, and CHMP4B in C6/36 cells (S9B Fig), indicating that prM did not participate in the recruitment of the ESCRT mechanism in the mosquito vector. However, mosTSG101 still regulates JEV replication in mosquito cells, independent of its interaction with prM, as evidenced by the fact that its knockdown inhibited, while its overexpression promoted, viral replication (Fig 8 N and 8O). In vertebrate hosts, TSG101 has been confirmed to be involved in the synthesis of JEV RNA and the assembly of viral particles. Using JEV replicon and VLP systems, we confirmed that mosTSG101 also supported viral RNA replication in mosquito hosts, evidenced by a significant attenuation of viral RNA synthesis in mosTSG101-knockdown C6/36 cells whereas a marked increase following its overexpression (Fig 8P and 8Q), which is consistent with results in vertebrate cells (Fig 7G). However, the assembly of viral particles was not interfered by the knockdown or overexpression of mosTSG101(Fig 8R). Thus, mosTSG101 plays an important role in regulating JEV replication in mosquito cells, which is independent on the ubiquitination of prM protein.

In summary, TSG101 acts as an adaptor bridging ubiquitylated prM and downstream ESCRTs to promote viral budding in vertebrate hosts, but not in mosquitoes.

## Discussion

As a pivotal post-translational modification, ubiquitination constitutes a crucial regulatory layer that controls protein structure, stability, intracellular localization, and interactions with other proteins [31]. Particularly, ubiquitination has garnered increasing attention for its roles in viral replication, acting as both antiviral and proviral factors [37,53]. This molecular tug-of-war is strategically leveraged by both hosts and viruses. Orthoflaviviruses have been proven to hijack the host ubiquitin system for efficient replication, in which the ubiquitination of viral proteins serves multiple functions in pathogenesis and the viral life cycle through both degradative and non-degradative mechanisms. Specifically, the ubiquitin-mediated degradation of viral proteins such as C [39], NS2A [40], and NS4A [42], is instrumental in promoting viral uncoating, driving infection, and enhancing pathogenicity, while non-degradative ubiquitination of viral proteins such as E [6,38], NS1 [41], and NS5 [44], is a critical determinant for virus entry, host adaptation, and host range. Nonetheless, the functional significance and molecular mechanism of viral protein ubiquitination for orthoflavivirus replication remain incomplete. Here, we identify JEV prM as a target for host-specific ubiquitination, which uniquely occurs in vertebrate hosts but not in mosquitoes. This modification is mediated by evolutionarily conserved lysine residues (K107/108/116) in multiple mosquito-borne orthoflaviviruses including USUV, MVEV, and WNV, and directly confers their differential adaptation to vertebrate hosts and the mosquito vector. Mechanistically, prM ubiquitination recruits the ESCRT-I subunit TSG101, which subsequently engages downstream components (VPS28, CHMP2A, and CHMP4B) to drive viral particle budding.

Compared to other orthoflavivirus, JEV has a broader spectrum of hosts, spanning multiple mammalian species (pigs, humans, horses, sheep, cattle, bats) [54–56], birds (migratory birds and waterfowl) [14,56,57], and mosquito vector [48]. However, the adaptability of JEV varies among different hosts. Despite high seroprevalence across numerous mammal and bird species [55,56], humans, pigs, and horses demonstrate a pronounced susceptibility to JEV, developing significant clinical symptoms with high viremia [58,59]. In contrast, infections in other species are typically sporadic and asymptomatic [60,61], suggesting critical differences in host-virus interactions at the molecular level. The ubiquitination of orthoflavivirus proteins is increasingly recognized as a pivotal mechanism governing their differential adaptation across host species. For instance, the host-specific NS5 ubiquitination determines the host tropism of YFV, ZIKV, and DENV to primates [44]. The ubiquitination of NS1 confers differential adaptation of ZIKV in mammalian hosts and mosquito vectors [41]. Here, we discovered a novel function of prM ubiquitination in species-specific support of JEV replication in vertebrate hosts *in vitro* and *in vivo*. Our results indicate that vertebrate-specific ubiquitination of prM protein is critical for JEV replication in vertebrate hosts (mice and ducklings), and its deficiency impairs replication, thereby reducing viremia and viral loads in tissue. Crucially, this ubiquitin-dependent mechanism is absent in the mosquito vector, highlighting the critical role of viral protein ubiquitination in underpinning the differential adaptation of JEV across species. Notably, constrained by the availability of current mosquito-borne animal models, this study used only *Aedes* mosquitoes as the infection vector, which may not adequately represent JEV infection dynamics in its natural *Culex* hosts. Given that *Culex* is the primary transmission vector [62], its inclusion would undoubtedly strengthen the robustness of our conclusions. We look forward to future investigations employing validated *Culex* models to further corroborate our current results.

Previous studies have reported that site-specific ubiquitination of orthoflavivirus prM proteins can affect viral replication [63,64], and these effects were commonly attributed to proteasomal degradation or destabilization of the modified viral proteins. Here, we uncover a novel function for prM ubiquitination that confers species-specific regulation of JEV replication and adaptation, thereby illustrating a distinct strategy by which the virus harnesses the host ubiquitin system to optimize its fitness. As arboviruses, orthoflavivirus actually also co-opted the ubiquitin system within mosquito vectors to enhance replication and pathogenicity. This is evidenced by the ubiquitination of viral proteins such as E [38], NS1 [41], and NS4A [42], which directly regulate infection dynamics within the mosquitoes. Nevertheless, divergent ubiquitination mechanisms between vertebrate hosts and mosquito vectors may result in opposing effects on orthoflavivirus replication. A striking example of this host-dependent opposition is the E2 conjugating enzyme Ubc9, which interacts with DENV E, NS1, and NS5 proteins to suppress replication in mammals yet promote it in mosquitoes [65]. Similarly, the E3 ligase WWP2 ubiquitinates ZIKV NS1, employing a degradative mechanism that exerts antiviral effects in mammalian hosts, whereas in mosquitoes, it employs a non-degradative mechanism to promote viral replication [41]. Furthermore, our study suggests that the host-specific ubiquitination of the prM protein, observed in vertebrates but absent in mosquito vectors, may also arise from the differences in ubiquitination mechanisms among various hosts. Given the importance of identifying common ubiquitination factors in viral infection for elucidating regulatory mechanisms and developing broad-spectrum antivirals, we initially attempted to screen for host-specific E3 ubiquitin ligases targeting prM proteins. Unfortunately, our initial screening in vertebrate hosts did not yield clear candidates, so the mechanism by which the prM protein undergoes specific ubiquitination in vertebrate hosts remains to be explored.

The classical ubiquitination pathway typically involves the attachment of a ubiquitin molecule to a lysine residue of a target protein [31]. In this study, we unraveled the first comprehensive landscape of site-specific ubiquitin modification in the JEV prM during infection. The combination of di-glycyl-targeted peptide enrichment and MS analysis resulted in the identification of three Ub-modified lysines (K107, K108, and K116) across prM, the majority of which have not been described nor functionally characterized before. Notably, the residues K107, K108, and K116 of prM are highly conserved in a specific clade of mosquito-borne orthoflavivirus phylogenetically related to JEV, including USUV, MVEV and WNV, and collectively contribute to the differential adaptation of these viruses to vertebrate hosts and mosquito vectors. These findings provided experimental evidence suggesting that K107, K108, and K116 sites are under evolutionary selection associated with host adaptation. Indeed, evolutionary selection of specific lysine residues in the prM protein has been demonstrated as a determinant of infectivity and virulence in mosquito-borne orthoflavivirus, such as in ZIKV [45,46]. Extending this paradigm, our study further defines residues K107, K108, and K116 as essential residues in the prM protein for JEV host tropism and neuroinvasiveness. This finding underscores the broad impact of the specific residue evolution within prM on orthoflavivirus pathogenesis, including host tropism and virulence.

Viral envelopment at the plasma membrane is an essential step in the formation of enveloped virus particles. Substantial evidence indicated that ubiquitination of viral proteins, exemplified by those of HCV [27,30], HIV [66], EBoV [67], and severe acute respiratory syndrome coronavirus 2 (SARS-CoV-2) [68], facilitated viral particle assembly and envelopment. Here, we found that the deficiency of prM protein ubiquitination inhibited viral envelopment, thereby leading to excessive accumulation of viral nucleocapsids in vertebrate hosts. This confirms that prM protein ubiquitination is specifically required for viral assembly, but not for other stages of the viral life cycle, including internalization, protein expression, RNA replication, and viral release. In orthoflavivirus, the prM protein is established as the principal driver of budding for viral envelope acquisition. Research has shown that the prM/E complex residing on the ER membrane is sufficient to induce budding and envelope acquisition independently of nucleocapsid or nonstructural proteins, thereby generating VLPs that are structurally analogous to virions [20,69]. Despite viral proteins orchestrating this process, its efficiency is heavily reliant on their interactions with host cellular factors. To acquire a viral envelope, viruses bud at the plasma membrane and/or intracellularly in a process that involves membrane curving and fission [22]. This budding topology, equivalent to that of vesicle budding into multivesicular bodies (MVBs), is mainly mediated by the ESCRT machinery [22,24]. Using mass spectrometry, we identified specific associations between the ubiquitinated prM protein and several ESCRT components-TSG101, VPS28, CHMPA, and CHMP4B. Functional studies demonstrated that knockdown or knockout of these proteins in vertebrate host cells significantly inhibits JEV assembly. Furthermore, TSG101 and VPS28 were also found to participate in viral RNA replication, indicating that the ESCRT machinery plays an essential role in the JEV replication cycle. Indeed, the ESCRT machinery has been previously implicated in infectious JEV production [30]. Correlative light electron microscopy has shown that a distinct set of ESCRT components (TSG101, CHMP2/3, and CHMP4 proteins) functions directly in membrane deformation during orthoflavivirus particle formation on the ER membrane [30], but their regulatory functions in viral assembly and recruitment mechanisms remain unclear. Here, our research reveals a mechanism wherein the ubiquitination of JEV prM protein mediates the recruitment of the ESCRT complex, thereby promoting viral assembly.

Ubiquitination of viral proteins serves as a recognition signal for ESCRT-dependent entry, thereby mediating virus budding [22,70]. For instance, HCV NS2 protein could interact with the ESCRT machinery via ubiquitination to facilitate viral envelopment [27], and the ubiquitin conjugation to Gag is required for ESCRT-mediated HIV-1 budding [66]. As the most upstream ESCRT component, TSG101 is essential for recruiting the ESCRT machinery to viral budding sites via its recognition of ubiquitinated cargo [50]. This recognition occurs through a conserved ubiquitin E2 variant (UEV) domain, which is structurally homologous to canonical E2 Ub-conjugating enzymes but lacks the catalytic cysteine that supports covalent Ub linkage [52]. Consistent with a report that TSG101 is recruited to orthoflavivirus ER budding sites as an adaptor for downstream ESCRTs [30], we found that TSG101 deficiency abrogated the recruitment of ESCRT components VPS28, CHMP2A, and CHMP4B by the ubiquitinated prM, resulting in approximately 40-fold viral titers reduction. Meanwhile, the disruption of TSG101 UEV domain significantly reduced its binding affinity for ubiquitinated prM, which in turn impaired the recruitment of downstream ESCRT components (VPS28, CHMP2A, CHMP4B). These results confirm that the recognition of ubiquitinated prM by the TSG101 UEV domain is essential for ESCRT recruitment and JEV assembly. Furthermore, our finding that VPS28, CHMP2A, and CHMP4B are also critical for viral assembly further underscores the collective action of multiple ESCRT factors in viral replication. Notably, TSG101 exhibits functional conservation in JEV-infected vertebrate cells, as both porTSG101 and duTSG101 homologs could effectively be recruited by ubiquitinated prM proteins to engage the downstream ESCRT machinery in BHK-21 cells. In contrast, mosquito-derived TSG101 cannot be recruited by prM in mosquito cells, and therefore failed to engage the downstream ESCRT mechanisms to regulate viral budding assembly. This functional deficit may be attributed to the ubiquitination deficiency of prM protein in mosquito vectors. Nonetheless, TSG101 still plays an essential role in JEV replication in mosquito cells in a prM-independent mechanism that remains to be elucidated.

**Fig 9:**
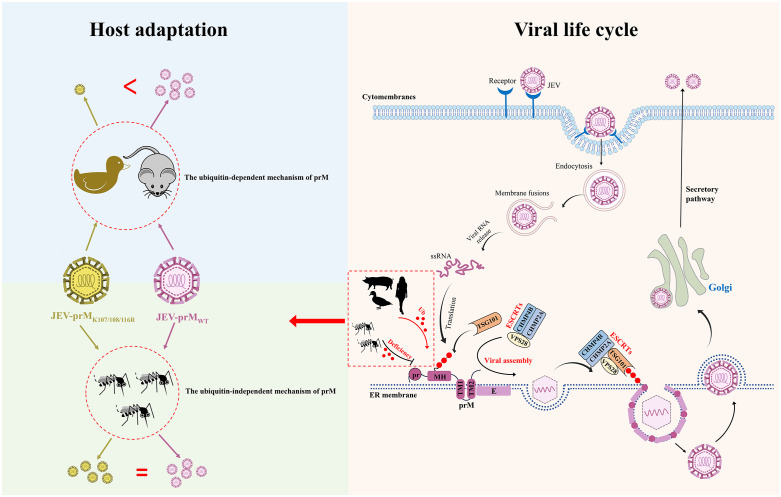
A model of prM protein ubiquitination contributes to the JEV adaptation in vertebrate hosts via recruiting ESCRT machinery to facilitate efficient viral assembly. In vertebrate hosts, prM ubiquitination functions as a recruitment signal for the ESCRT-I subunit TSG101, an early-acting ESCRT, which in turn acts as an adaptor to enlist downstream ESCRT components (VPS28, CHMP2A, and CHMP4B), thereby driving viral particle budding.

In summary, we found that JEV prM protein undergoes a significant ubiquitination in vertebrate hosts, but not in the mosquito vector. The host-specific ubiquitination of prM confers differential adaptation of JEV in vertebrate hosts and mosquito vectors. Mechanistically, prM ubiquitination functions as a recruitment signal for the ESCRT-I subunit TSG101, which further functions as an adaptor to recruit downstream ESCRT components (VPS28, CHMP2A, and CHMP4B), thereby driving viral particle budding (Fig 9). These results elucidate a novel mechanism of ubiquitination within the viral protein in regulating JEV infection and host adaptation, and provide potential implications for the design of novel antiviral strategies against orthoflaviviruses.

## Materials and methods

### Ethical statements and biosafety procedures

All the animal experiments were performed according to the Regulations of Jiangsu Province Laboratory Animal Management, and approved by the Institutional Animal Care and Use Committee of Yangzhou University (Mouse: IACUC No. 202202174; Duck: IACUC No. 202507017). All biological experiments for JEV in this study were approved by the Institutional Biosafety Committee (IBC) of Yangzhou University and conducted in a Biosafety Level 2 (BSL-2) facility, in strict accordance with all relevant biosafety guidelines and regulations.

### Cells and viruses

BHK-21, HEK-293T, ST, DF-1, and S2 cells were purchased from American Type Culture Collection (ATCC, Manassas, VA, USA). PIEC and C6/36 cells were purchased from Wuhan Pricella Biotechnology Co., Ltd (Wuhan, China). DEF was prepared from 11- to 13-day-old SPF duck embryos (Harbin Veterinary Research Institute, Chinese Academy of Agricultural Sciences, Harbin, China). Cxq-1, a *Culex* cell line derived from newly hatched larval tissues of *Culex pipiens quinquefasciatus*, was kindly gifted by Professor Feng Cui, Institute of Zoology, Chinese Academy of Sciences. BHK-21, HEK-293T, ST, DF-1, PIEC and DEF cells were cultured in Dulbecco’s modified Eagle’s medium (DMEM; Cytiva, Marlborough, MA, USA) with 10% fetal bovine serum (FBS; Sigma, St. Louis, MO, USA), 100 μg/ml streptomycin and 100 IU/ml penicillin (Cytiva) at 37˚C. C6/36 cells were cultured using RPMI 1640 medium (Cytiva) with 10% FBS at 28°C with no additional CO_2_. The *Drosophila melanogaster* S2 cells were cultured in Schneider’s medium (Gibco, Carlsbad, CA) supplemented with 10% FBS at 28°C with no additional CO_2_. The Cxq-1 cells were cultured in M199 medium (Sigma) supplemented with 10% FBS and 0.5% lactalbumin hydrolysate (Sigma) at 28°C with no additional CO_2_. For the following viral infection, all cells were maintained in a medium containing 2% FBS, 100 μg/mL streptomycin, and 100 IU/mL penicillin. The wild-type JEV virulent strain Beijing/2020–1 (GenBank No: OP588746.1) isolated from mosquitoes [6], and the DTMUV virulent strain TA (GenBank: JQ289550.1) isolated from a dead duck with egg drop disease [71], were stored in our laboratory and propagated in BHK-21 cells.

### Antibodies and reagents

Rabbit monoclonal anti-JEV E protein, mouse monoclonal anti-JEV C protein, rabbit polyclonal anti-JEV prM protein, and mouse monoclonal anti-JEV NS1’ protein antibodies were purchased from GeneTex (St. Anthony, TX, USA). Mouse monoclonal anti-DTMUV E protein antibody [71] was kindly donated by Prof. Yun Zhang (Harbin Veterinary Research Institute, Chinese Academy of Agricultural Sciences). Mouse monoclonal anti-ubiquitin, rabbit monoclonal anti-ubiquitin, rabbit monoclonal anti-TSG101, rabbit monoclonal anti-Calnexin, and rabbit monoclonal anti-CHMP4B antibodies were purchased from Cell Signaling Technology (CST; Danvers, MA, USA). Rabbit polyclonal anti-CHMP2A, and rabbit monoclonal anti-VPS28 were purchased from Proteintech (Wuhan, China). Mouse monoclonal anti-Flag, rabbit monoclonal anti-Flag, mouse monoclonal anti-HA and rabbit polyclonal anti-HA were purchased from ABclonal (Woburn, MA, USA). Rabbit polyclonal anti β-tubulin antibody was purchased from Bioworld Technology (Dublin, OH, USA). HRP-conjugated goat anti-mouse and HRP-conjugated goat anti -rabbit antibodies were purchased from BBI Life Science (Shanghai, China). Alexa Fluor 488-AffiniPure goat anti-mouse IgG(H + L), Alexa Fluor 488-AffiniPure goat anti-rabbit IgG (H + L), Alexa Fluor 647-AffiniPure goat anti-rabbit IgG(H + L), and Alexa Fluor 647-AffiniPure goat anti-mouse IgG (H + L) were purchased from Jackson ImmunoResearch (PA, USA). Anti-Flag magnetic beads and anti-HA magnetic beads were purchased from Selleck Chemical (Houston, TX, USA). The Flag-nanobody magnetic beads were purchased from AlpalifeBio (Guangzhou, China). The inhibitors TAK-243 and MG132 were purchased from MedChemExpress (MCE; NJ, USA).

### Virus growth kinetics

To assess virus growth kinetics, cells pre-seeded in 24-well plates to 90% confluence were respectively infected with JEV, DTMUV, chimeric viruses (rGI-USUV/Flag-prM, rGI-WNV/Flag-prM, rGI-MVEV/Flag-prM, rDTMUV-Flag-prM), and their corresponding mutants at a multiplicity of infection (MOI) of 0.05. Following infection, culture supernatants were collected at designated time points. Infectious virus titers in the supernatants were subsequently determined by titration on BHK-21 cells and calculated as log_10_TCID_50_ (median tissue culture infective dose) per 0.1 mL using the Reed-Muench method.

### Generation of mutated and chimeric viruses

The reverse genetics system for JEV (strain Beijing/2020–1; pOK-rGI) has been constructed and stored in our lab [6,72]. Site-directed mutagenesis of the ubiquitination sites (K107, K108 and K116) within prM of the JEV infectious cDNA clone pOK-rGI (rGI/Beijing/2020–1) was performed by overlap PCR. The primers used are listed in S3 Table. For viral rescues, the JEV cDNA clones were first linearized with the restriction endonuclease *SalI*, and then utilized to transcribe full-length viral RNA using the mMessage mMachine T7 kit (Invitrogen; MA, USA). The generated viral RNA was transfected into BHK-21 cells in six-well plates with DMRIE-C reagent (Invitrogen). At 2–3 days post-transfection, culture supernatants of the transfectants exhibiting typical cytopathic effects (CPE) of JEV infection were harvested and amplified in BHK-21 cells.

The chimeric viruses rGI-USUV/Flag-prM, rGI-WNV/Flag-prM or rGI-MVEV/Flag-prM were generated by incorporating the structural proteins (C, prM with an N-terminal Flag-tag, and E) from USUV (GenBank No. KX601691.1), WNV (GenBank No. JN183891.1) or MVEV (GenBank No. NC000943.1), respectively, into the nonstructural protein backbone (NS1-NS5) of the JEV strain Beijing/2020–1.

Briefly, the JEV infectious cDNA clone pOK-rGI was modified to remove the structural proteins C, prM, and E using the restriction endonucleases *NotI* and *AgeI*. The coding sequences of the structural proteins of USUV, WNV, or MVEV with a Flag-tag at the N-terminus of the prM proteins synthesized by GenScript Biotechnology (Nanjing, China) were cloned into JEV infectious clones through homologous recombination, respectively. To ensure the accessibility of the Flag-tag and avoid interfering with the processing and function of prM protein, the Flag-tag was flanked by the first four residues of JEV prM at the N-terminus and a short flexible Gly-Ser-Gly linker at the C-terminus. Site-directed mutagenesis of the ubiquitination sites (K107, K108, and K116) within prM of USUV, WNV, or MVEV was performed by overlap PCR. The primers used are listed in S3 Table. The rescue of viruses was performed as described above [6].

For DTMUV, a PCR-based reverse genetics system was utilized to construct mutated viruses or recombinant virus rDTMUV-Flag-prM [73]. Four pairs of primers targeting the highly conserved sequences of the DTMUV TA strain (S3 Table) were designed to amplify four overlapping fragments covering the full-length viral genome. Four overlapping cDNA segments (F1: 1–2406, F2: 2366–5092, F3: 5025–8318, and F4:8259–10991) of DTMUV were inserted into the pOK12 vector to generate four recombinant plasmids (pDTMUV-I, pDTMUV-II, pDTMUV-III and pDTMUV-IV. The full-length cDNA corresponding to the entire viral genome was generated by a series of PCRs using four recombinant plasmids as templates. To generate the recombinant DTMUV with Flag-tagged prM, two overlapping fragments (nucleotides 1–507 and 467–2406) were amplified by PCR with a primer pair containing the Flag-tag and linker sequences using pDTMUV-I as a template and spliced through homologous recombination to generate the recombinant plasmid, pDTMUV-I-prM-Flag. Subsequently, pDTMUV-I-prM-Flag together with pDTMUV-II, pDTMUV-III and pDTMUV-IV were utilized to generate recombinant virus rDTMUV-Flag-prM. The rescue of viruses was performed as described above [6].

### JEV VLP secretion assay

To produce JEV virus-like particles (VLPs), the prM-E gene expression cassette, incorporating the C-terminal capsid-derived signal peptide for correct prM-E translocation and processing, was amplified and cloned into vector pOK-12. To enhance translational efficiency, the coding sequence was flanked by 5’ and 3’ UTRs of JEV, and preceded by a T7 promoter for *in vitro* RNA transcription.

Equal doses of VLP RNA and its mutants were transfected into BHK-21 cells, respectively. At 36 hours post-transfection (hpt), the cell culture supernatant was collected, and then proteins contained therein were precipitated by adding polyethylene glycol (PEG-6000) to a final concentration of 20% (w/v) and incubating at 4°C for 18 hours. Subsequently, the precipitate was pelleted by centrifugation at 10,000 × g for 1 hour at 4°C, and the retained proteins were subjected to sucrose density gradient centrifugation to purify JEV VLPs. The content of JEV VLPs was analyzed by SDS-PAGE.

### The *Gaussia* luciferase assay

The JEV replicon and the recombinant reporter JEV expressing a *gaussia* luciferase gene (rGI-GLuc) were constructed as previously described [73,74]. The JEV replicon RNA, the reporter virus RNA or their corresponding mutants were transfected into cells, and the culture supernatants of transfected cells were harvested at designated time points post-transfection. The luciferase activity was determined in a luminometer (LumiStation-1800) by mixing 20 μL culture supernatant with 50 μL reaction substrate Coelenterazine h (20 μm, pH 7.2; Maokang Biotechnology, Shanghai, China).

### Mass spectrometry analysis of ubiquitination sites

BHK-21 cells were infected with JEV strain Beijing/2020–1 at an MOI of 1. At 24 hours post-infection (hpi), cells were treated with MG132 (5 μM) for 4 h, and then lysed in RIPA buffer (50 mm Tris [pH 7.6], 150 mm NaCl, 1% NP-40, 0.5% sodium deoxycholate). The cell lysate was subjected to immunoprecipitation by incubating with rabbit anti-prM antibody-conjugated beads for 6 hours at 4°C. Subsequently, the enriched JEV prM was used for ubiquitination mass spectrometry analysis based on K-ε-GG (di-glycyl) remnant antibody enrichment, which involves digestion to peptides, immunoaffinity enrichment with an antibody recognizing di-glycine remnants left behind at ubiquitinated lysines, and liquid chromatography-tandem mass spectrometry analysis of the enriched peptides.

### The identification of ubiquitinated prM-interacting proteins by mass spectrometry

BHK-21 cells were transfected with the equivalent amounts of viral RNA derived from either rGI-Flag-prM or its ubiquitination-deficient mutant rGI-Flag-prM-K107/108/116R. At 12 hpt, cells were immunoprecipitated with Flag-nanobody magnetic beads. The immunoprecipitated proteins were separated by SDS-PAGE and stained with Coomassie Brilliant Blue. The protein bands specifically associated with the ubiquitinated prM were excised and subjected to in-gel tryptic digestion. The resulting peptides were subsequently analyzed by liquid chromatography-mass spectrometry (LC-MS/MS) at Qinglianbio Biotechnology (Beijing, China)

### Ubiquitination assay

Cells were transfected with plasmids expressing either Flag-prM or its mutants, together with or without HA-tagged ubiquitin (HA-Ub). At 36 hpt, the cells were lysed in RIPA buffer and then subjected to immunoprecipitation with anti-Flag or anti-HA magnetic beads. The ubiquitination of Flag-prM was then assessed by Western blot using anti-HA and anti-Flag antibodies.

To investigate prM ubiquitination upon viral infection, a panel of viruses including rGI, several Flag-prM tagged chimeras (rDTMUV-Flag-prM, rGI-USUV/Flag-prM, rGI-WNV. Flag-prM, rGI-MVEV/Flag-prM), and their corresponding mutants was utilized to infect various cell lines at an MOI of 1. At 24 hpi (HEK-293T, ST, and DEF) or 60 hpi (C6/36), the whole-cell lysates were immunoprecipitated with the anti-prM or anti-Flag antibody-conjugated beads and thereafter probed for ubiquitination by Western blot with anti-Ub antibody.

### Neuroinvasiveness and neurovirulence assays in mice

Three-week-old weanling female C57BL/6 mice (five mice per group) purchased from the Laboratory Animal Center of Yangzhou University were inoculated intraperitoneally (100 μL/each) or intracerebrally (10 μL/each) with either rGI or rGI-K107/108/116R at doses of 10^3^ and 10^5^ TCID_50_ to measure neuroinvasiveness, or at doses ranging from 100 to 1000 TCID_50_ to measure neurovirulence, respectively. Mice were monitored daily for mortality and clinical signs of disease. The survival curve was calculated by the method of Reed and Muench using GraphPad Prism 8.0.

### Viral host adaptation in mice and ducklings

Three-week-old weanling female C57BL/6 mice (five mice per group) purchased from the Laboratory Animal Center of Yangzhou University and two-day-old domestic ducklings (ten ducklings per group) purchased from Hanchao Poultry Co., Ltd. (Xiaoshan District, Hangzhou, China), were intraperitoneally mock-inoculated with PBS or inoculated with 10^5^ TCID_50_ of rGI or rGI-K107/108/116R, respectively. At the designated time points post-infection, the infected mice and ducklings were euthanized to collect blood samples and various tissues. The levels of viremia and viral loads in tissues were measured by TCID_50_ assay, as described previously [6,11].

### Viral host adaptation in mosquitoes

Female *Aedes aegypti* mosquitoes, aged 5–7days post-emergence, were purchased from KePu Biotechnology Company (Lianyungang, China). All mosquitoes were maintained on 10% sucrose ad libitum solution and reared at 28°C with 80% relative humidity in accordance with standard rearing procedures. For blood feeding, fresh mouse blood was collected in heparin-coated tubes and centrifuged at 1,000g for 10 minutes at 4 °C to separate plasma from blood cells. The plasma was heat-inactivated at 55 °C for 60 minutes, and then recombined with the blood cells. Mosquitoes were pre-starved for 16–24 hours, and then fed with a mixture of the treated blood and virus supernatant containing 10^6^ TCID_50_/mL of rGI or rGI-K107/108/116R, using an artificial feeding system [54] (Hemotek, Lancashire, UK). After a 40-minute feeding period, the engorged female mosquitoes were separated into new containers and kept for at least one week to analyze the infection ratios. The whole mosquito (n = 20), salivary gland (n = 20), and midgut (n = 20) were collected at 7, 10, and 14 days post-infection for viral determination using TCID_50_ assays as previously [75].

For intrathoracic inoculation, mosquitoes with similar body size were anesthetized on ice and intrathoracically injected with rGI or rGI-K107/108/116R at a dose of 500 TCID_50_ (0.5μL per mosquito) with an Eppendorf Cell Tram oil microinjector (Hamburg, Germany), as described previously [61]. The whole mosquito (n = 20), salivary gland (n = 20), and midgut (n = 20) were collected at 7, 10, and 14 days post-infection for the viral titration using TCID_50_ assays.

### Immunoblotting and indirect immunofluorescence assay

Immunoblotting analysis was performed as previously described [73]. Briefly, the whole cell lysates or immunoprecipitates were separated by 10% SDS-PAGE and subsequently transferred onto a nitrocellulose (NC) membrane. The membrane was blocked with 5% skim milk in PBS, and then incubated with primary and secondary antibodies. The visualization of protein bands was performed using the Tanon 5200 Multi chemiluminescent imaging system (Tanon).

For the indirect immunofluorescence assay, cells were fixed with 4% paraformaldehyde for 1 h, permeabilized in 0.05% NP-40 for 15 min, and blocked with 5% bovine serum albumin (BSA) at 37°C for 1 h. Subsequently, cells were incubated with primary antibody and corresponding secondary antibodies, each for 1 hour at room temperature. The nuclei were counterstained with 4,6-diamidino-2-phenylindole (DAPI; Solarbio, Beijing, China) for 15 min. Finally, fluorescent images were captured using a fluorescence microscope (Olympus).

### shRNA-mediated knockdown of gene expression

For gene silencing, sense and antisense DNA oligonucleotides encoding shRNA (S4 Table) targeting TSG101, VPS28, CHMP2A and CHMP4B mRNA were synthesized, annealed, and inserted into the pLKO.1-Puro vector. Lentivirus expressing shRNAs was produced by co-transfecting HEK-293T cells with the recombinant pLKO.1-shRNA construct, along with the packaging plasmids pCMV-VSV-G and psPAX2. At 48 hpt, the supernatant containing lentiviral particles was collected and filtered through 0.45 µm filters. Target cells were infected with the lentivirus for 48 h, and stable knockdown cell lines were selected with puromycin (3 µg/mL). Knockdown efficiency was confirmed by Western blotting.

### CRISPR-Cas9-based genome editing

TSG101-KO BHK-21 were generated using the CRISPR/Cas9 editing method. The guide RNA targeting mouse TSG101 DNA (5′-AGAGTCAGCTGAAGAAGATG -3′) was designed using the E-CRISP online tool (http://www.e-crisp.org/E-CRISP/designcrispr.html). The gene knockout cells with green fluorescence were sorted into 96-well plates by FACS and confirmed by immunoblotting and sequencing the genomic DNA.

### RNA quantification

Total viral RNA was extracted with TRIzol reagent (Vazyme, Nanjing, China) from cell lysates, and then reverse-transcribed to cDNA using a cDNA synthesis kit (Vazyme). Quantitative real-time PCR (qPCR) was performed using the SYBR Green qPCR amplification kit (Vazyme) on a QuantGene 9600 system (Bioer, Hangzhou, China) to quantify viral genomes. The sequences of primers used are listed in S3 Table.

### Phylogenetic analysis and sequence alignment

The prM protein sequence of the representative viruses within the genus *orthoflavivirus,* including JEV (GenBank No. OP588746.1), Usutu Virus (USUV, GenBank No. KX601691.1), Murray Valley encephalitis virus (MVEV, GenBank No. NC000943.1), WNV (GenBank No. JN183891.1), Kunjin virus (KUNV, GenBank No. AY274505.1), DTMUV (GenBank No. KY810818.1), Saint Louis encephalitis virus (SLEV, GenBank No. KM267635.1), ZIKV (GenBank No. OL450366.1), DENV (GenBank No. MG599631.1), Yellow fever virus (YFV, GenBank No. JX949181.1), Karshi virus (KSIV, GenBank No. AY863002.1), Omsk hemorrhagic fever virus (OHFV, GenBank No. OP037815.1), TBEV (GenBank No. OP037819.1), Alkhurma hemorrhagic fever virus (AHFV, GenBank No. JX271893.1), Deer tick virus (DTV, GenBank No. OP037825.1) and Powassan virus (POWV, GenBank No. PQ788268.1), were retrieved from NCBI database (http://www.ncbi.nlm.nih.gov) and phylogenetically analyzed with MEGA version 12 software (https://www.megasoftware.net). The multiple sequence alignment was performed with the DNASTAR Software (DNAStar, Madison, WI, USA).

### Homology modeling

The mouse TSG101 was modeled with SWISS-MODEL (https://swissmodel.expasy.org/). The structure of TSG101 UEV domain (Protein Data Bank accession number 1KPP) was visualized and analyzed using PyMOL (http://www.pymol.org) and SPDBV (DeepView) software ([href:https://spdbv.unil.ch/]https://spdbv.unil.ch/).

### Statistical analyses

Statistical analyses were performed using GraphPad Prism software (version 8.0.1, San Diego, CA, USA). Quantitative data were represented as means ± standard deviations (SD) from at least three independent experiments. A *p*-value < 0.05 was considered statistically significant.

## Supporting information

S1 Table Secondary mass spectrometry analysis of ubiquitination of prM. (DOCX)

S2 Table The result of mass spectrometry analysis. (XLSX)

S3 Table The primers used for PCR and qPCR in this study. (DOCX)

S4 Table The shRNAs used for gene silencing in this study. (DOCX)

S1 Fig Ubiquitination of JEV prM protein in BHK-21, PIEC, DF-1, and S2cells. (A-D) The ubiquitination analysis of JEV prM protein in BHK-21 (A), PIEC (B), DF-1 (C), and S2 (D) cells. Cells were transfected with a plasmid expressing Flag-prM, along with a plasmid expressing HA-Ub or an empty vector. At 36 hpt, cell lysates were harvested for immunoprecipitation assays using anti-Flag or anti-HA magnetic beads. The ubiquitination of prM protein was analyzed by immunoblotting with an anti-HA or anti-Flag antibody. (E-H) The ubiquitination analysis of the JEV prM protein in JEV-infected BHK-21 (E), PIEC (F), DF-1 (G), and S2 (H) cells. Cells were infected with the JEV virulent strain Beijing/2020–1 at an MOI of 1. At 24 hpi (BHK-21, PIEC, and DF-1) or 60 hpi (S2), cell lysates were harvested for immunoprecipitation assays using a prM antibody and analyzed by Western blotting using an anti-ubiquitin antibody.
(TIF)

S2 Fig Overall ubiquitination levels upon viral infection and NS1 ubiquitination in C6/36 cells. (A) C6/36 cells infected with the JEV virulent strain Beijing/2020–1 (MOI = 1) were harvested at 48 and 60 hpi, and the overall ubiquitination levels in the lysates were analyzed by Western blotting using an anti‑ubiquitin antibody. (B) The ubiquitination analysis of JEV prM and NS1 protein in C6/36 cells. Cells were transfected with a plasmid expressing Flag-prM or Flag-NS1, along with a plasmid expressing HA-Ub or an empty vector. At 36 hpt, cell lysates were harvested for immunoprecipitation assays using anti-Flag magnetic beads. The ubiquitination of prM and NS1 protein was analyzed by immunoblotting with an anti-HA antibody.
(TIF)

S3 Fig Generation and verification of chimeric viruses. (A, B) Immunofluorescence analysis of WT chimeric viruses or their mutants in BHK-21 cells. Scale bar: 200 μm. JEV NS1’ protein, DTMUV E protein, Flag-prM, and nuclei were stained with anti-NS1’ antibody (red), Anti-E antibody (red), anti-Flag antibody (green), and DAPI (blue), respectively. (C) Western blot analysis of JEV NS1’, DTMUV E and Flag-prM in cells infected with WT JEV, WT DTMUV, or chimeric viruses. The cellular β-tubulin was used as a loading control. (D) The rescued viruses were serially passaged five times in BHK-21 cells and validated by Sanger sequencing.
(TIF)

S4 Fig The ubiquitination of JEV prM protein is involved in viral assembly in ST cells. (A-C) Assessment of viral attachment in ST cells incubated with rGI or rGI-K136R-K166R at 4°C for 1 h, by IFA (A), Western blotting (B), and RT-qPCR (C). (D) Internalization analysis of ST cells infected with JEV at an MOI of 10 by qPCR. (E) Luciferase assay of ST cells transfected with equal doses of rGI-GLuc or rGI-GLuc-K107/108/116R RNA. (F, G) Sucrose density-gradient analysis of intracellular viral particles derived from ST cells transfection with equal doses of rGI or rGI-K107/108/116R RNA. Fourteen fractions were collected and subjected to measurement of the nucleocapsid and intracellular particle by immunoblotting (F) and TCID_50_ assays (G). (H) The total viral titer from supernatant and cell lysates in ST cells. (I, J) The extracellular (I) and intracellular virus titers (J) in ST cells. (K) Comparison of extracellular and total infectious viral titers. Data are shown as means ± SD (C-E and G-K). Statistical analysis was performed using the unpaired Student’s *t*-test. ***p < 0.001, ns, no significance.
(TIF)

S5 Fig Generation and verification of recombinant viruses rGI-Flag-prM and rGI-Flag-prM-K107/108/116R. (A) Immunofluorescence analysis of rGI-Flag-prM and rGI-Flag-prM-K107/108/116R in BHK-21 cells. Scale bar: 200 μm. JEV NS1’ protein, Flag-prM, and nuclei were stained with anti-NS1’ antibody (red), anti-Flag antibody (green), and DAPI (blue), respectively. (B) The rescued virus was serially passaged five times in BHK-21 cells and validated by Sanger sequencing. (C) Western blot analysis of JEV NS1’ and Flag-prM in cells infected with rGI or rGI-Flag-prM. The cellular β-tubulin was used as a loading control. (D) The growth kinetics of rGI, rGI-Flag-prM, rGI-prM-K107/108/116R and rGI-Flag-prM-K107/108/116R in BHK-21 cells.
(TIF)

S6 Fig Mass spectrometry identification of TSG101, VPS28, CHMP2A or CHMP4B as potential interacting partners of ubiquitinated prM. Red indicates matched B ions, blue indicates matched Y ions, and grey indicates precursor ions.
(TIF)

S7 Fig The interaction between prM and ESCRT protein in JEV-infected BHK-21 cells with the overexpression of TSG101 or the knock-down of VPS28, CHMP2A, or CHMP4B. (A) Immunoprecipitation analysis of prM protein and its interacting cellular proteins in TSG101-overexpressed BHK-21 cells infected with rGI-Flag-prM or rGI-Flag-prM-K107/108/116R. (B-D) Immunoprecipitation analysis of the interaction between prM and ESCRT protein in BHK-21 cells upon infection with rGI-Flag-prM or rGI-Flag-prM-K107/108/116R, with or without knockdown of VPS28 (B), CHMP2A (C), and CHMP4B (D).
(TIF)

S8 Fig The intensity profile of the linear region of interest (ROI) across the BHK-21 cells costained with prM and TSG101. (TIF)

S9 Fig The interactions of prM and mosTSG101 with ESCRT components in C6/36 and BHK-21 cells. (A) BHK-21 cells were transfected with the plasmid expressing Myc-mouTSG101 or Myc-mosTSG101 for 36 h, and then lysed for immunoprecipitation with an anti-Myc antibody. The protein complex was analyzed by immunoblotting. (B) C6/36 cells with the overexpression of mosVPS28, mosCHMP2A or mosCHMP4B were respectively infected with rGI-Flag and rGI-Flag-prM-K107/108/116R at an MOI of 1. At 36 hpt, cells were harvested for immunoprecipitation assays using anti-Flag magnetic beads. The protein complex was analyzed by immunoblotting.
(TIF)
