# Supplementary material for: Host-specific ubiquitination of prM orchestrates ESCRT recruitment to mediate efficient Japanese Encephalitis Virus assembly in vertebrates
Source: PLoS Pathog. 2026 Jul 8;22(7):e1014426. doi: 10.1371/journal.ppat.1014426 (PMC13362398; doi:10.1371/journal.ppat.1014426)

| Sequence | Modifications | Positions in Master Proteins | Modifications in Master Proteins |
| --- | --- | --- | --- |
| CWVRAIDVGYMCEDTITYECPK | 1xGG [K22] | target [34-55] | target 1xGG [K55] |
| KEAWLDSTK | 1xGG [K1] | target [108-116] | target 1xGG [K108] |
| EAWLDSTKATR | 1xGG [K8] | target [109-119] | target 1xGG [K116] |
| KEAWLDSTKATRYLMK | 1xGG [K16] | target [108-123] | target 1xGG [K123] |
| KEAWLDSTKATRYLMK | 2xGG [K1; K9] | target [108-123] | target 2xGG [K108; K116] |
| SVSVHTHGESSLVNKKEAWLDSTK | 2xGG [K15; K16] | target [93-116] | target 2xGG [K107; K108] |

S1 Table. Secondary mass spectrometry analysis of ubiquitination of prM


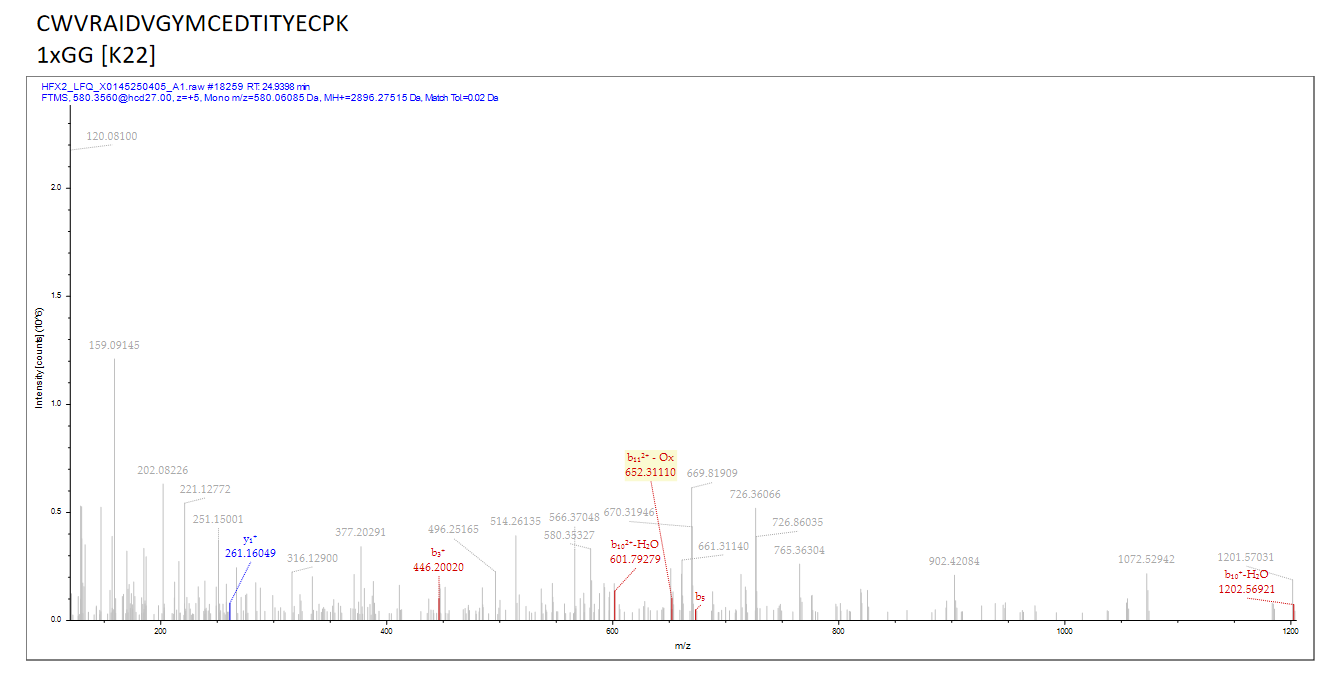

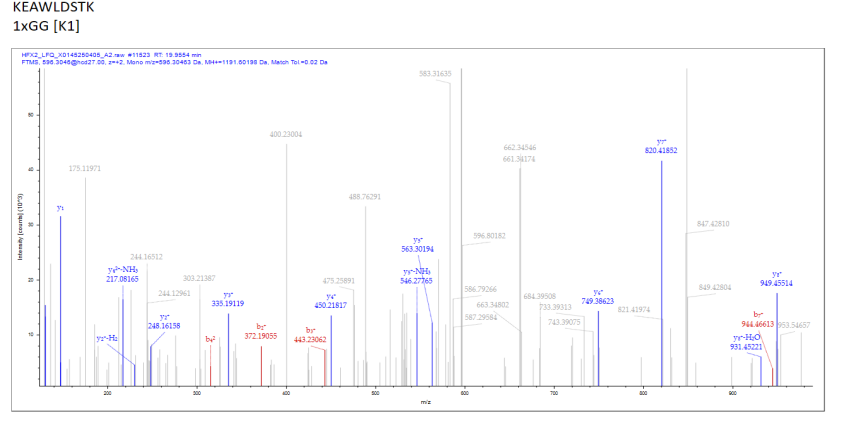

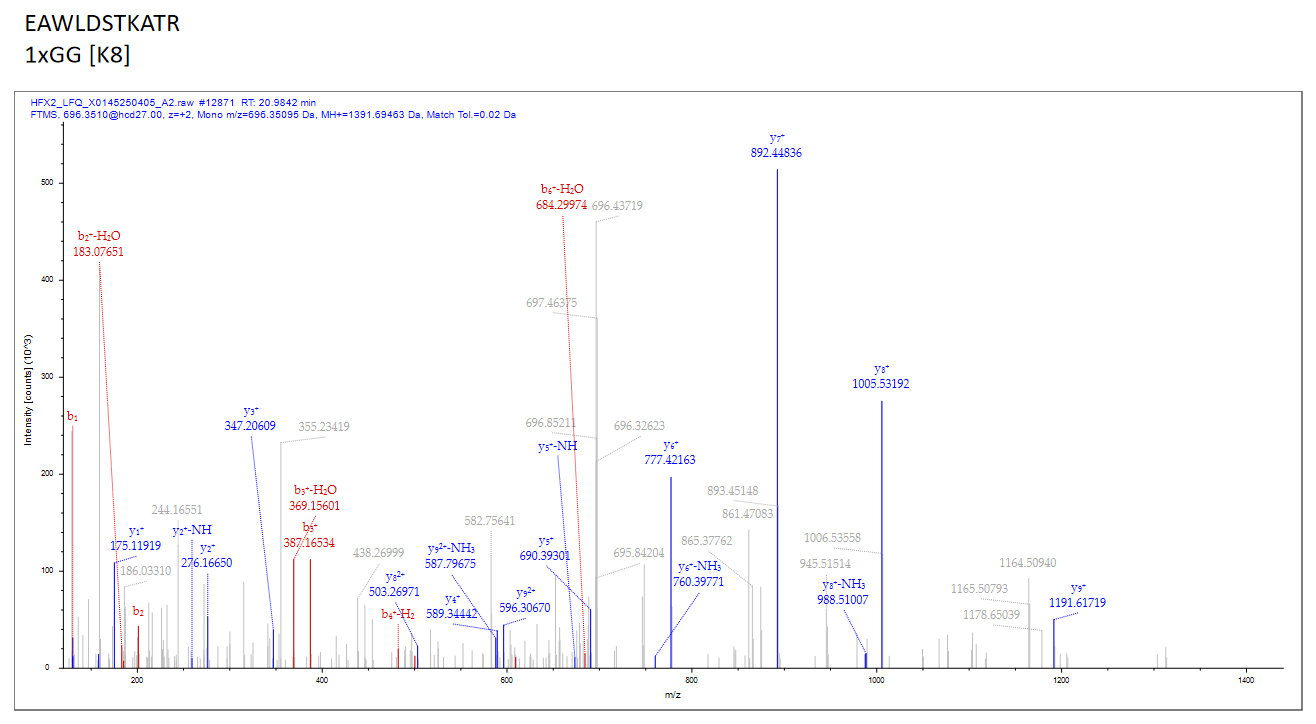

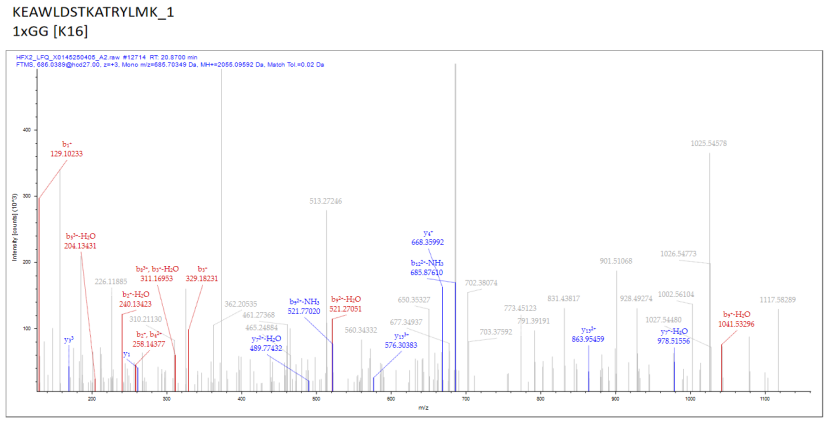

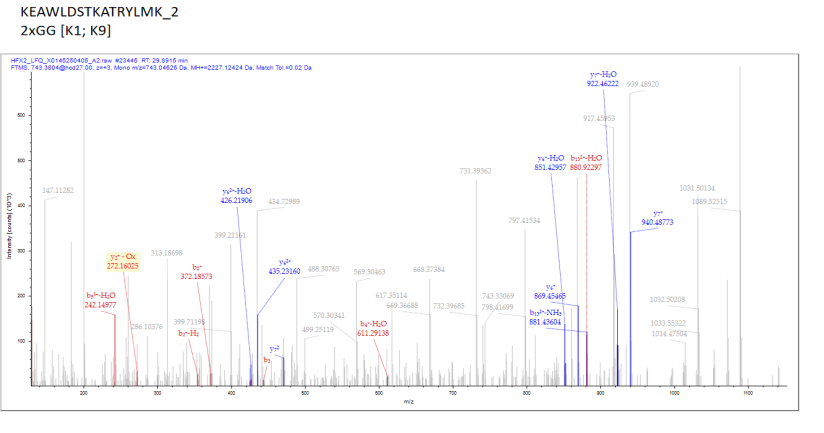

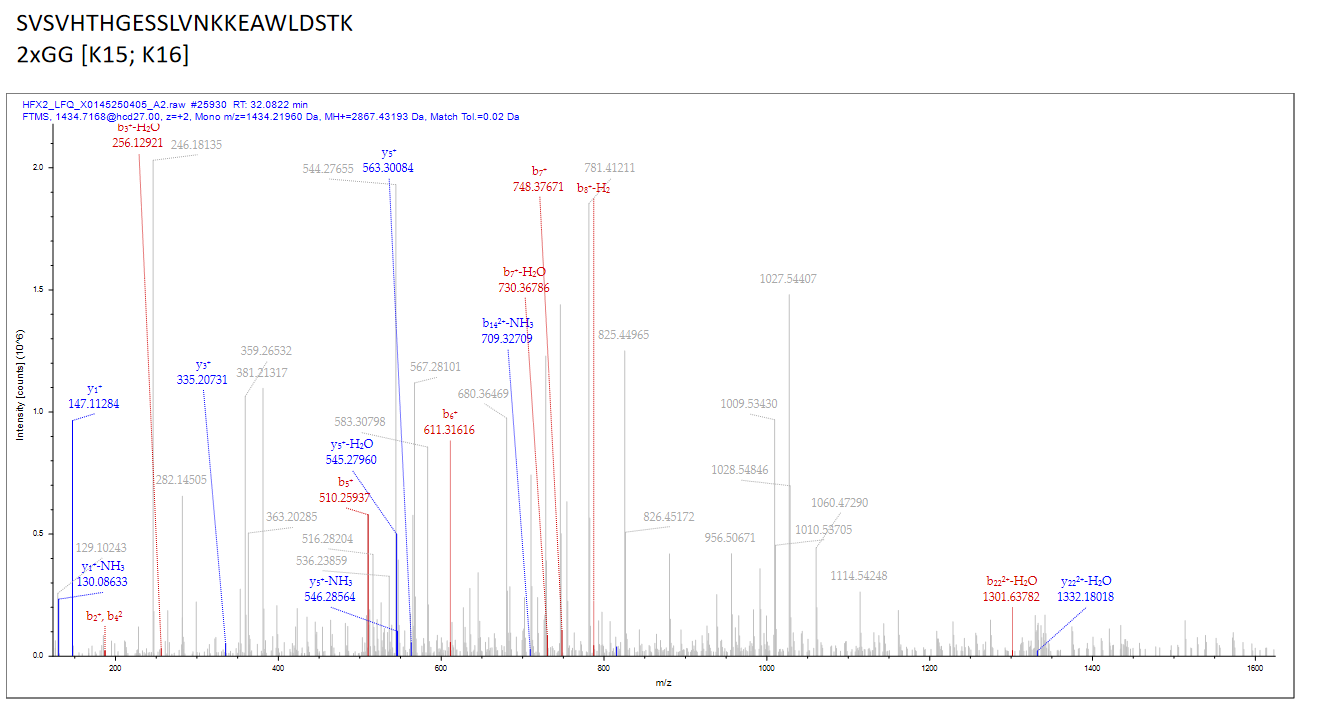


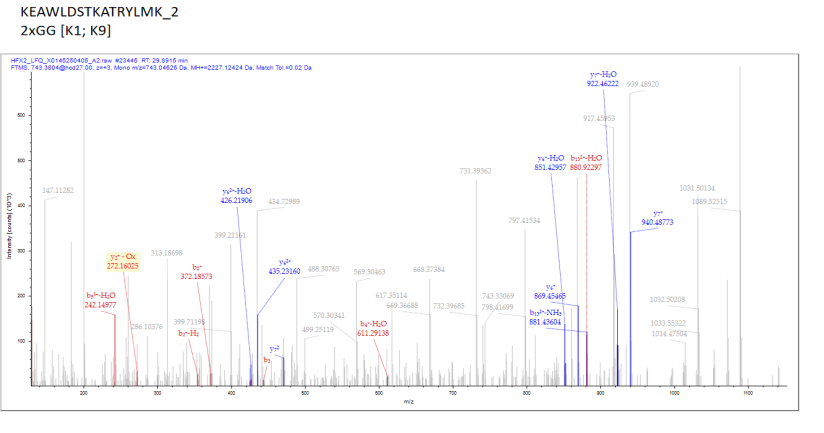

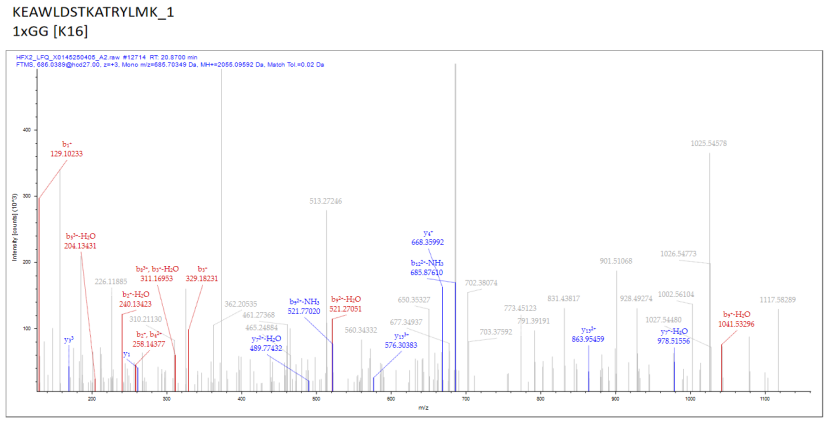

Supplement: S1 Table — (DOCX) [file ppat.1014426.s001.docx]
