# Supplementary material for: Host-specific ubiquitination of prM orchestrates ESCRT recruitment to mediate efficient Japanese Encephalitis Virus assembly in vertebrates
Source: PLoS Pathog. 2026 Jul 8;22(7):e1014426. doi: 10.1371/journal.ppat.1014426 (PMC13362398; doi:10.1371/journal.ppat.1014426)
Supplement: S3 Table — (DOCX) [file ppat.1014426.s003.docx]

| Plasmids | PCR Primers (5'-3')  S3 Table. The primers used for PCR and qPCR in this study |
| --- | --- |
| pOK-rGI-K107R | F:CTAGTGAACAGAAAAGAGGCTTGG |
|  | R:CCAAGCCTCTTTTCTGTTCACTAG |
| pOK-rGI-K108R | F:TCACTAGTGAACAAAAGAGAGGCTTGG |
|  | R:CCAAGCCTCTCTTTTGTTCACTAGTGA |
| pOK-rGI-K116R | F:CTGGATTCAACGAGGGCCACGCGATACC |
|  | R:GGTATCGCGTGGCCCTCGTTGAATCCAG |
| pOK-rGI-K107/108R | F:CTAGTGAACAGAGAAGAGGCTTGG |
|  | R:CCAAGCCTCTTCTCTGTTCACTAG |
| pOK-rGI-MVEV-K107/108/116R  or  MVEV-Flag-prM-K107/108/116R | F:AAAGCACTTTGGTCAACAGAAGGGATGCCTGGCTGGATTCCACGAGGGCCACGCGTTAT |
|  | R:ATAACGCGTGGCCCTCGTGGAATCCAGCCAGGCATCCCTTCTGTTGACCAAAGTGCTTT |
| pOK-rGI-USUV-K107/108/116R  or  USUV-Flag-prM-K107/108/116R | F:GAGTATGCTGGCTAACAGGAGGGATGCTTGGCTAGACTCAACCAGGGCTTCGAGATACC |
|  | R:GGTATCTCGAAGCCCTGGTTGAGTCTAGCCAAGCATCCCTCCTGTTAGCCAGCATACTC |
| pOK-rGI-WNV-K107/108/116R  or  WNV-Flag-prM-K107/108/116R | F:CTCTAGCGAACAGGAGGGGGGCTTGGATGGACAGCACCAGGGCCACAAGGTA |
|  | R:TACCTTGTGGCCCTGGTGCTGTCCATCCAAGCCCCCCTCCTGTTCGCTAGAG |
| DTMUV-Flag-prM-K107/108/116R | F:GTCTACTTGAGGCCAGGAACACGCCGTGGATGGATTCGACCAGAGCCACTAAATATCTC |
|  | R:GAGATATTTAGTGGCTCTGGTCGAATCCATCCACGGCGTGTTCCTGGCCTCAAGTAGAC |
| pDTMUV-I | F:GCTAATACGACTCACTATAGGAGAAGTTCATCTGTGTGAACTTATTCCAAACAGCTTTT |
|  | R:TCATAGAAATGGATCTGTCCCTTGCATTCAGCCCCATC |
| pDTMUV-II | F:GTGGATGGGGCTGAATGCAAGGGACAGATCCATTTCTA |
|  | R:CCTTGAGTGATCATACTGACAAAATCGCCTGAACCCACCAGGA |
| pDTMUV-III | F:TGGTTGGCCTCTATGGTAATGGAATCCTGGTGGGTTCAGG |
|  | R:CCAGTACATCTCATGTGTTGAATTACGCGAGAGTGGCACCCTA |
| pDTMUV-IV. | F:TGGGGAGGTGGTTTGGTTAGGGTGCCACTCTCGCGTAAT |
|  | R:AGACTCTGTGTTCTACCACCACCAGCCACACTTTCGGCGATCTG |
| rDTMUV-Flag-prM | F1:ACGACGATAAGGGCTCAGGCCTGAAGCTTGGAAACTATAATGGTAGAGTTTTGG |
|  | F2:GGCTGAAGCTTGGAGATTACAAGGATGACGACGATAAGGGCTCAGGCCTGAAGCTTGGA |
|  | R:CTTATCGTCGTCATCCTTGTAATCTCCAAGCTTCAGCCCAGCAACTATCGGGAGTAA |
| Gene name | qPCR primers (5'-3') |
| JEV | F:GGGTCAGATCCGTCACTAGAC |
|  | R:ACGACGAACGTGGAGTTGGC |
