# Supplementary material for: Host-specific ubiquitination of prM orchestrates ESCRT recruitment to mediate efficient Japanese Encephalitis Virus assembly in vertebrates
Source: PLoS Pathog. 2026 Jul 8;22(7):e1014426. doi: 10.1371/journal.ppat.1014426 (PMC13362398; doi:10.1371/journal.ppat.1014426)
Supplement: S4 Table — (DOCX) [file ppat.1014426.s004.docx]

| Proteins  S4 Table. The shRNAs used for gene silencing in this study | Sequences (5'-3') |
| --- | --- |
| Mouse TSG101 | shRNA-F: CCAAATACCTCCTACATGCCAGGTCAAGAGCCTGGCATGTAGGAGGTATTTGGTTTTTT |
|  | shRNA-R: AAAAAACCAAATACCTCCTACATGCCAGGCTCTTGACCTGGCATGTAGGAGGTATTTGG |
| Mouse VPS28 | shRNA-F: GCCGAAAGTTCAGACTGGACTGCTCAAGAGGCAGTCCAGTCTGAACTTTCGGCTTTTTT |
|  | shRNA-R: AAAAAAGCCGAAAGTTCAGACTGGACTGCCTCTTGAGCAGTCCAGTCTGAACTTTCGGC |
| Mouse CHMP2A | shRNA-F: TGTTACTAAGGCCATGGGCACTATCAAGAGTAGTGCCCATGGCCTTAGTAACATTTTTT |
|  | shRNA-R: AAAAAATGTTACTAAGGCCATGGGCACTACTCTTGATAGTGCCCATGGCCTTAGTAACA |
| Mouse CHMP4B | shRNA-F: TGCTCAAGAACATGGGCTATGCCTCAAGAGGGCATAGCCCATGTTCTTGAGCATTTTTT |
|  | shRNA-R: AAAAAATGCTCAAGAACATGGGCTATGCCCTCTTGAGGCATAGCCCATGTTCTTGAGCA |
