# Supplementary figures and images for: Host-specific ubiquitination of prM orchestrates ESCRT recruitment to mediate efficient Japanese Encephalitis Virus assembly in vertebrates
Source: PLoS Pathog. 2026 Jul 8;22(7):e1014426. doi: 10.1371/journal.ppat.1014426 (PMC13362398; doi:10.1371/journal.ppat.1014426)

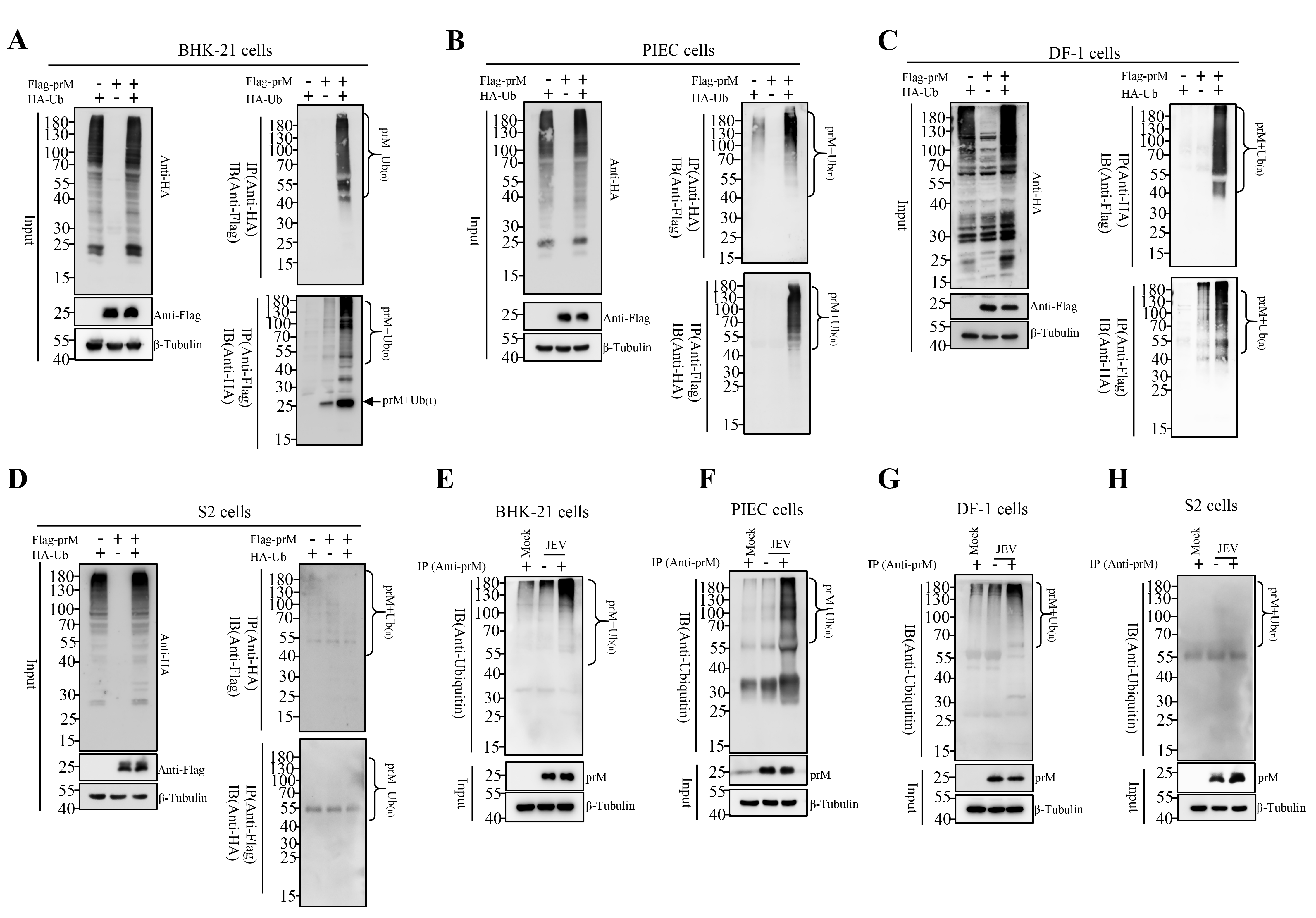

Supplement: S1 Fig — (A-D) The ubiquitination analysis of JEV prM protein in BHK-21 (A), PIEC (B), DF-1 (C), and S2 (D) cells. Cells were transfected with a plasmid expressing Flag-prM, along with a plasmid expressing HA-Ub or an empty vector. At 36 hpt, cell lysates were harvested for immunoprecipitation assays using anti-Flag or anti-HA magnetic beads. The ubiquitination of prM protein was analyzed by immunoblotting with an anti-HA or anti-Flag antibody. (E-H) The ubiquitination analysis of the JEV prM protein in JEV-infected BHK-21 (E), PIEC (F), DF-1 (G), and S2 (H) cells. Cells were infected with the JEV virulent strain Beijing/2020–1 at an MOI of 1. At 24 hpi (BHK-21, PIEC, and DF-1) or 60 hpi (S2), cell lysates were harvested for immunoprecipitation assays using a prM antibody and analyzed by Western blotting using an anti-ubiquitin antibody. (TIF) [file ppat.1014426.s005.tif]

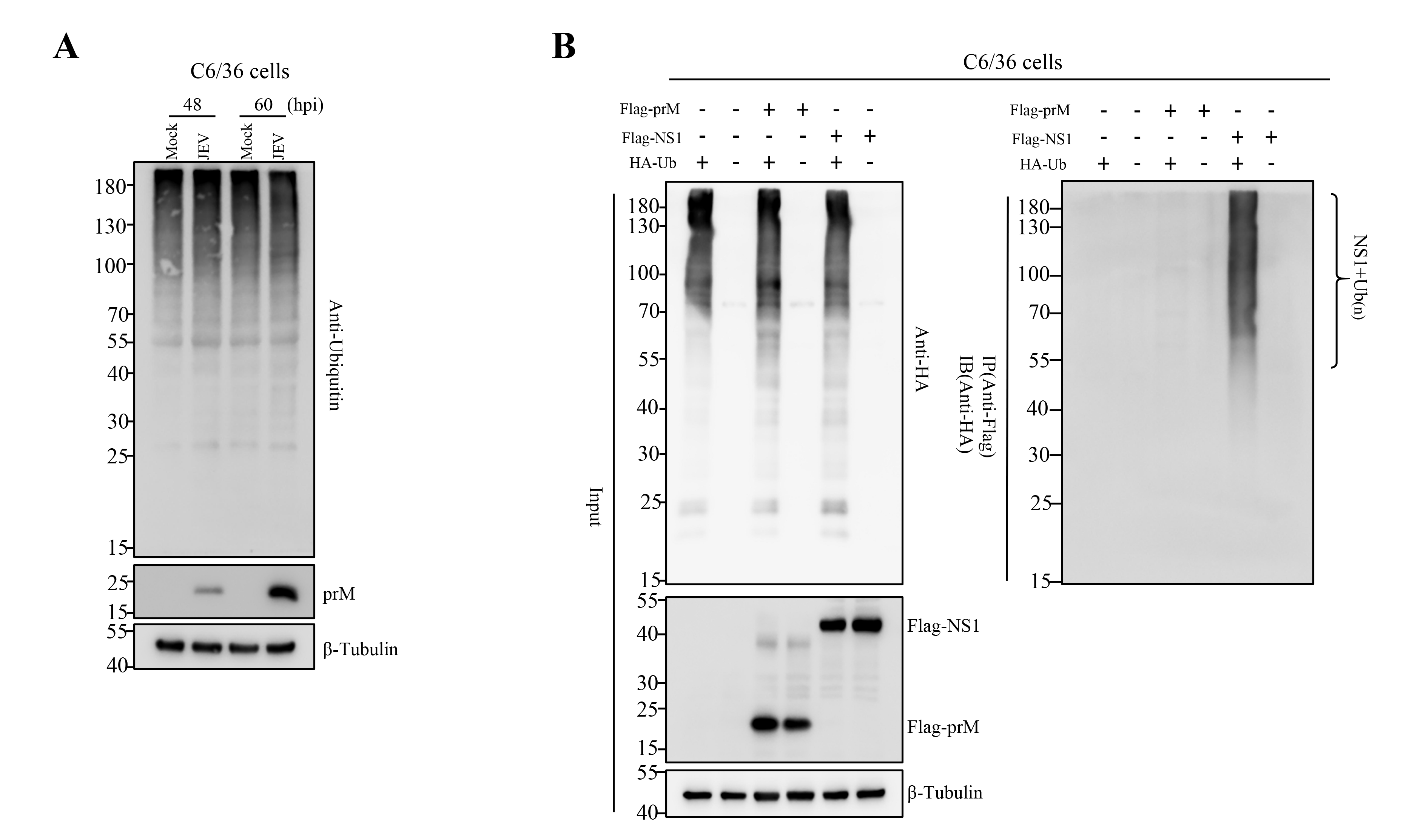

Supplement: S2 Fig — (A) C6/36 cells infected with the JEV virulent strain Beijing/2020–1 (MOI = 1) were harvested at 48 and 60 hpi, and the overall ubiquitination levels in the lysates were analyzed by Western blotting using an anti‑ubiquitin antibody. (B) The ubiquitination analysis of JEV prM and NS1 protein in C6/36 cells. Cells were transfected with a plasmid expressing Flag-prM or Flag-NS1, along with a plasmid expressing HA-Ub or an empty vector. At 36 hpt, cell lysates were harvested for immunoprecipitation assays using anti-Flag magnetic beads. The ubiquitination of prM and NS1 protein was analyzed by immunoblotting with an anti-HA antibody. (TIF) [file ppat.1014426.s006.tif]

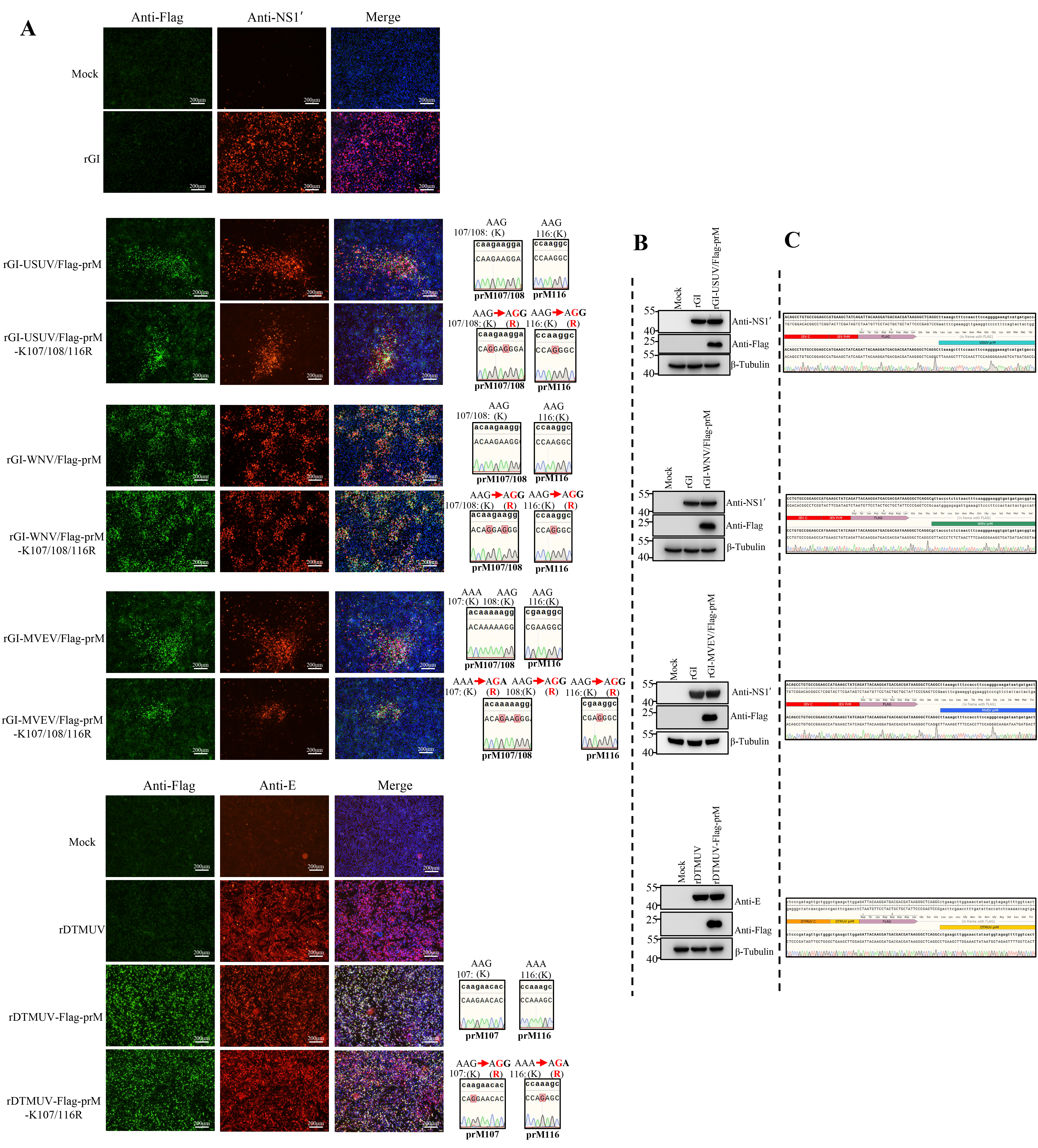

Supplement: S3 Fig — (A, B) Immunofluorescence analysis of WT chimeric viruses or their mutants in BHK-21 cells. Scale bar: 200 μm. JEV NS1’ protein, DTMUV E protein, Flag-prM, and nuclei were stained with anti-NS1’ antibody (red), Anti-E antibody (red), anti-Flag antibody (green), and DAPI (blue), respectively. (C) Western blot analysis of JEV NS1’, DTMUV E and Flag-prM in cells infected with WT JEV, WT DTMUV, or chimeric viruses. The cellular β-tubulin was used as a loading control. (D) The rescued viruses were serially passaged five times in BHK-21 cells and validated by Sanger sequencing. (TIF) [file ppat.1014426.s007.tif]

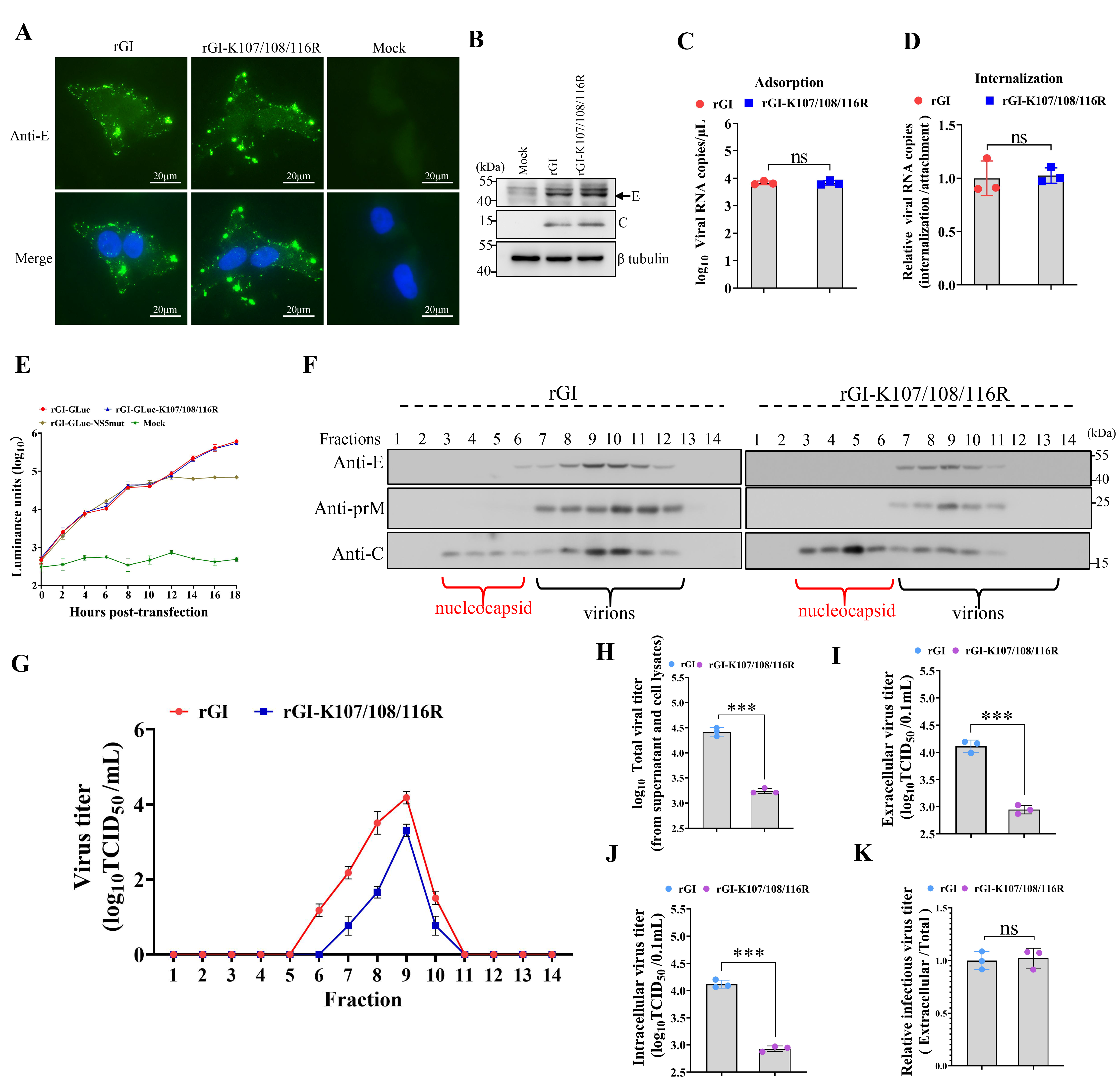

Supplement: S4 Fig — (A-C) Assessment of viral attachment in ST cells incubated with rGI or rGI-K136R-K166R at 4°C for 1 h, by IFA (A), Western blotting (B), and RT-qPCR (C). (D) Internalization analysis of ST cells infected with JEV at an MOI of 10 by qPCR. (E) Luciferase assay of ST cells transfected with equal doses of rGI-GLuc or rGI-GLuc-K107/108/116R RNA. (F, G) Sucrose density-gradient analysis of intracellular viral particles derived from ST cells transfection with equal doses of rGI or rGI-K107/108/116R RNA. Fourteen fractions were collected and subjected to measurement of the nucleocapsid and intracellular particle by immunoblotting (F) and TCID50 assays (G). (H) The total viral titer from supernatant and cell lysates in ST cells. (I, J) The extracellular (I) and intracellular virus titers (J) in ST cells. (K) Comparison of extracellular and total infectious viral titers. Data are shown as means ± SD (C-E and G-K). Statistical analysis was performed using the unpaired Student’s t-test. ***p < 0.001, ns, no significance. (TIF) [file ppat.1014426.s008.tif]

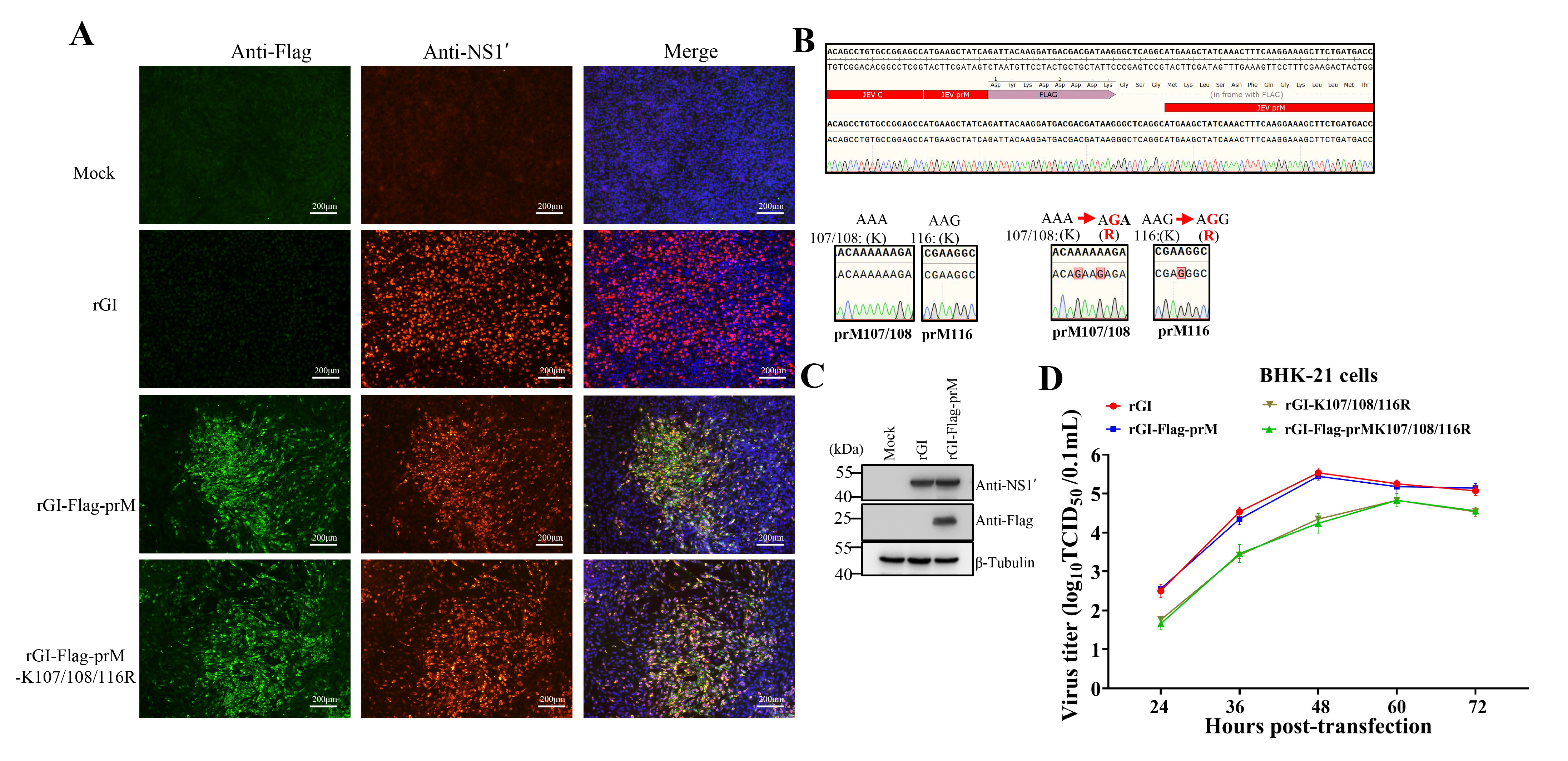

Supplement: S5 Fig — (A) Immunofluorescence analysis of rGI-Flag-prM and rGI-Flag-prM-K107/108/116R in BHK-21 cells. Scale bar: 200 μm. JEV NS1’ protein, Flag-prM, and nuclei were stained with anti-NS1’ antibody (red), anti-Flag antibody (green), and DAPI (blue), respectively. (B) The rescued virus was serially passaged five times in BHK-21 cells and validated by Sanger sequencing. (C) Western blot analysis of JEV NS1’ and Flag-prM in cells infected with rGI or rGI-Flag-prM. The cellular β-tubulin was used as a loading control. (D) The growth kinetics of rGI, rGI-Flag-prM, rGI-prM-K107/108/116R and rGI-Flag-prM-K107/108/116R in BHK-21 cells. (TIF) [file ppat.1014426.s009.tif]

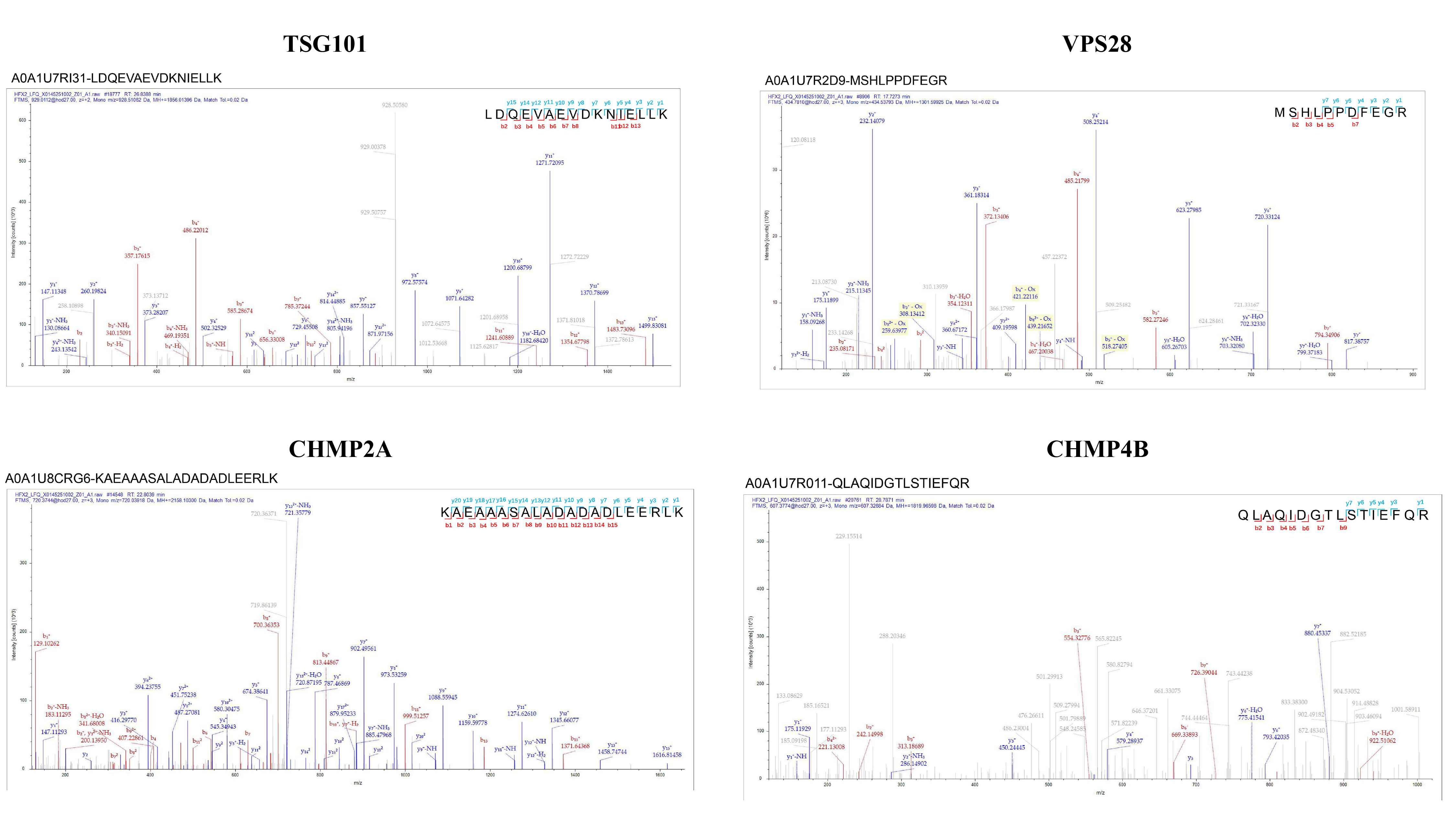

Supplement: S6 Fig — Red indicates matched B ions, blue indicates matched Y ions, and grey indicates precursor ions. (TIF) [file ppat.1014426.s010.tif]

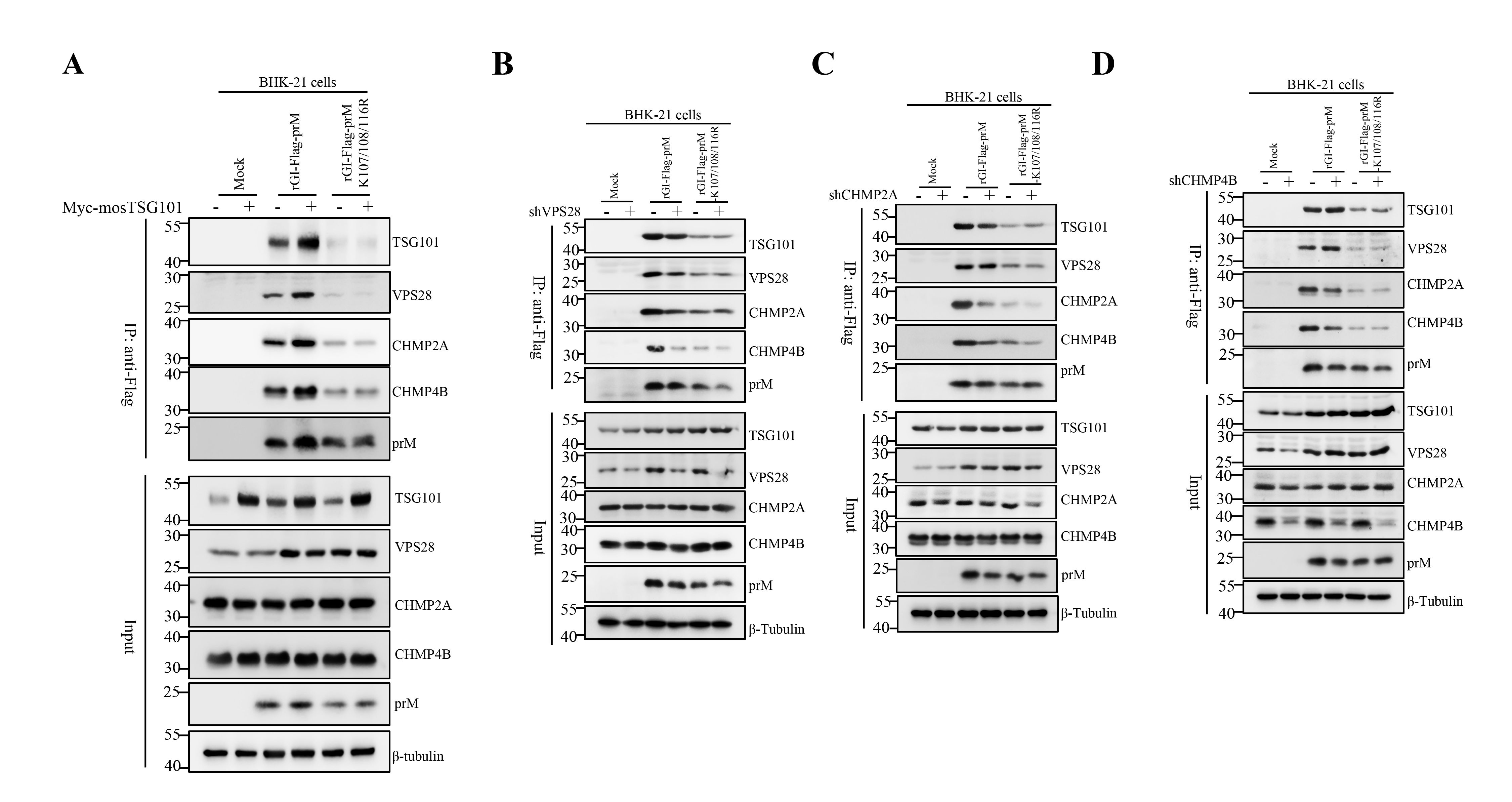

Supplement: S7 Fig — (A) Immunoprecipitation analysis of prM protein and its interacting cellular proteins in TSG101-overexpressed BHK-21 cells infected with rGI-Flag-prM or rGI-Flag-prM-K107/108/116R. (B-D) Immunoprecipitation analysis of the interaction between prM and ESCRT protein in BHK-21 cells upon infection with rGI-Flag-prM or rGI-Flag-prM-K107/108/116R, with or without knockdown of VPS28 (B), CHMP2A (C), and CHMP4B (D). (TIF) [file ppat.1014426.s011.tif]

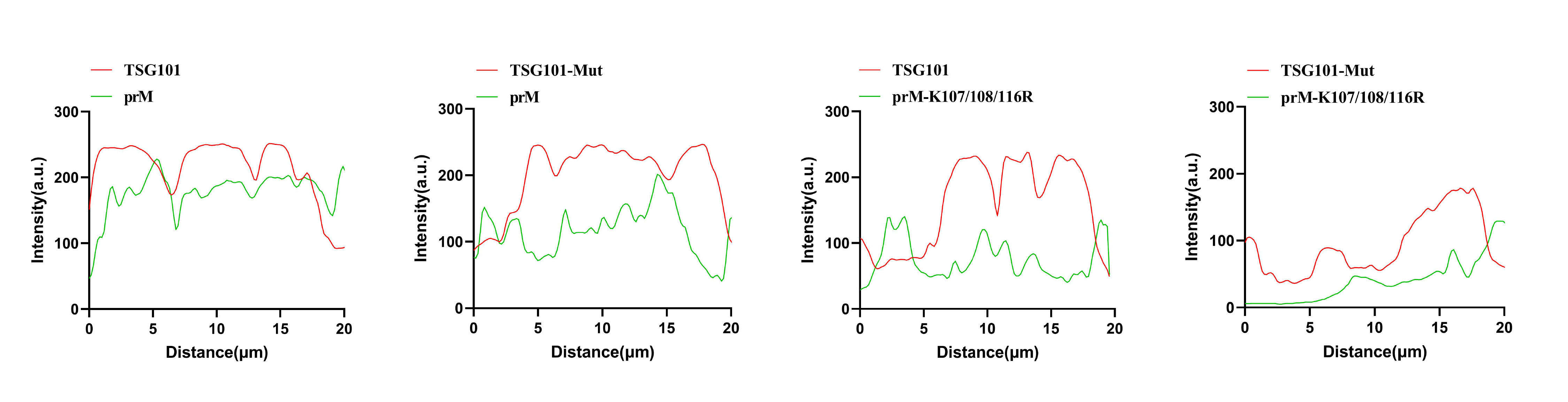

Supplement: S8 Fig — (TIF) [file ppat.1014426.s012.tif]

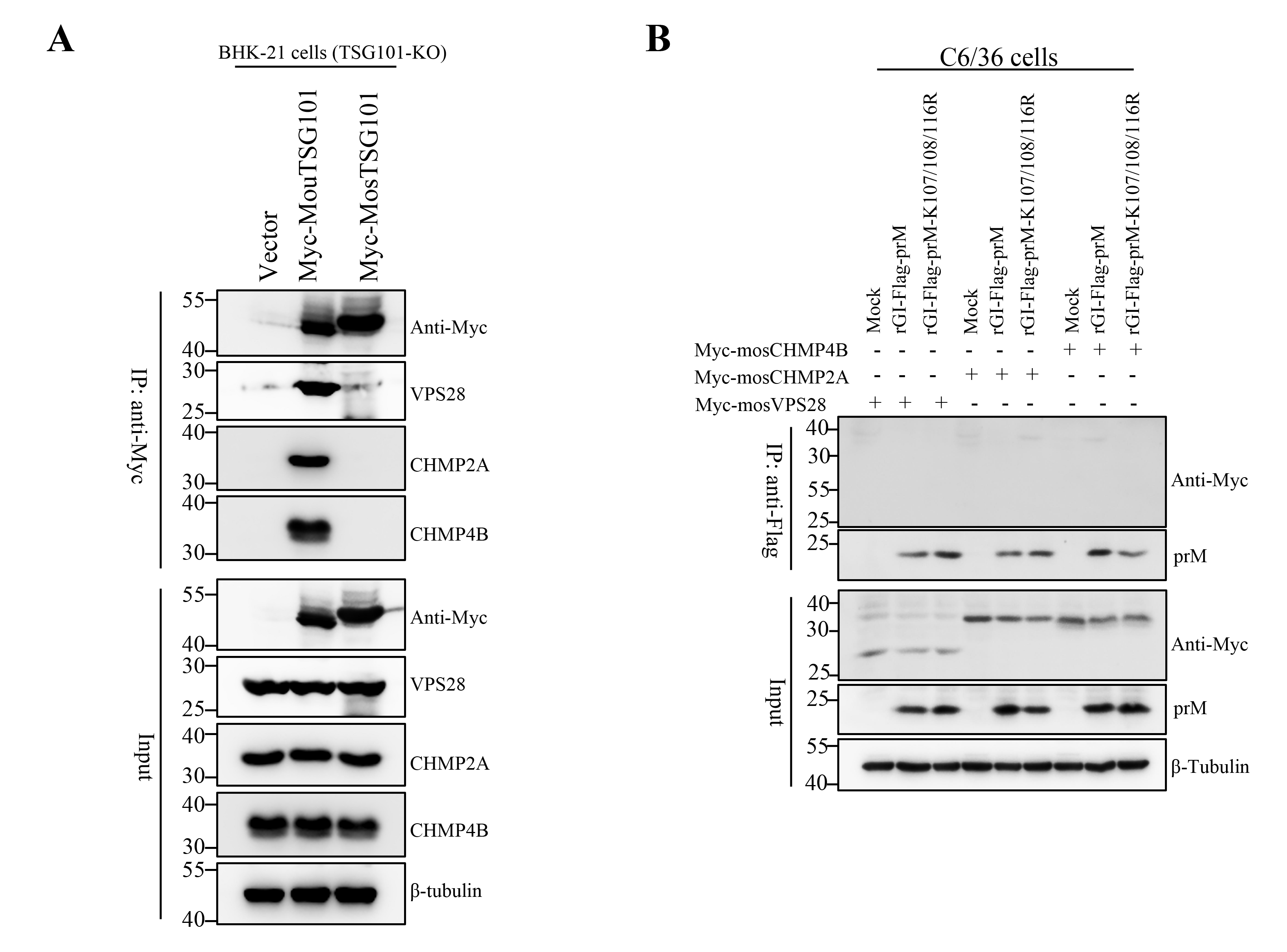

Supplement: S9 Fig — (A) BHK-21 cells were transfected with the plasmid expressing Myc-mouTSG101 or Myc-mosTSG101 for 36 h, and then lysed for immunoprecipitation with an anti-Myc antibody. The protein complex was analyzed by immunoblotting. (B) C6/36 cells with the overexpression of mosVPS28, mosCHMP2A or mosCHMP4B were respectively infected with rGI-Flag and rGI-Flag-prM-K107/108/116R at an MOI of 1. At 36 hpt, cells were harvested for immunoprecipitation assays using anti-Flag magnetic beads. The protein complex was analyzed by immunoblotting. (TIF) [file ppat.1014426.s013.tif]
